# Determining Excitatory and Inhibitory Neuronal Activity from Multimodal fMRI Data Using a Generative Hemodynamic Model

**DOI:** 10.3389/fnins.2017.00616

**Published:** 2017-11-10

**Authors:** Martin Havlicek, Dimo Ivanov, Alard Roebroeck, Kamil Uludağ

**Affiliations:** Department of Cognitive Neuroscience, Faculty of Psychology and Neuroscience, Maastricht University, Maastricht, Netherlands

**Keywords:** excitatory-inhibitory, multi-modal data, fMRI signal modeling, response transients, neuronal adaptation, post-stimulus BOLD undershoot, hemodynamic uncoupling, DCM

## Abstract

Hemodynamic responses, in general, and the blood oxygenation level-dependent (BOLD) fMRI signal, in particular, provide an indirect measure of neuronal activity. There is strong evidence that the BOLD response correlates well with post-synaptic changes, induced by changes in the excitatory and inhibitory (E-I) balance between active neuronal populations. Typical BOLD responses exhibit transients, such as the early-overshoot and post-stimulus undershoot, that can be linked to transients in neuronal activity, but they can also result from vascular uncoupling between cerebral blood flow (CBF) and venous cerebral blood volume (venous CBV). Recently, we have proposed a novel generative hemodynamic model of the BOLD signal within the dynamic causal modeling framework, inspired by physiological observations, called P-DCM (Havlicek et al., 2015). We demonstrated the generative model's ability to more accurately model commonly observed neuronal and vascular transients in single regions but also effective connectivity between multiple brain areas (Havlicek et al., 2017b). In this paper, we additionally demonstrate the versatility of the generative model to jointly explain dynamic relationships between neuronal and hemodynamic physiological variables underlying the BOLD signal using multi-modal data. For this purpose, we utilized three distinct data-sets of experimentally induced responses in the primary visual areas measured in human, cat, and monkey brain, respectively: (1) CBF and BOLD responses; (2) CBF, total CBV, and BOLD responses (Jin and Kim, 2008); and (3) positive and negative neuronal and BOLD responses (Shmuel et al., 2006). By fitting the generative model to the three multi-modal experimental data-sets, we showed that the presence or absence of dynamic features in the BOLD signal is not an unambiguous indication of presence or absence of those features on the neuronal level. Nevertheless, the generative model that takes into account the dynamics of the physiological mechanisms underlying the BOLD response allowed dissociating neuronal from vascular transients and deducing excitatory and inhibitory neuronal activity time-courses from BOLD data alone and from multi-modal data.

## Introduction

Functional magnetic resonance imaging (fMRI) is a widely used non-invasive technique to assess brain function. The fMRI signal reflects neuronal activity only indirectly through the measurements of accompanying hemodynamic processes at temporal resolution typically on the order of seconds and spatial resolution typically on the order of tens of cubic millimeters. In general, neuronal activation causes a series of physiological events, including localized changes in cerebral blood flow (CBF), cerebral metabolic rate of oxygen (CMRO_2_), cerebral blood volume (CBV), and deoxyhemoglobin content. These physiological variables form the basis of the blood oxygenation level-dependent (BOLD) signal (Ogawa et al., 1990), the most commonly used fMRI approach. However, CBF and CBV can also be directly measured with various fMRI techniques (e.g., see Wong et al., 1997; Lu et al., 2003; Liu and Brown, 2007; Huber et al., 2014b).

In many studies, it has been found that there is high correspondence between response properties measured in fMRI and other hemodynamic techniques and those measured from invasive electrical recordings, mostly acquired in non-human primates, cats and rodents (Logothetis et al., 2001, 2010; Kim et al., 2004; Niessing et al., 2005; Devor et al., 2007, 2013; Logothetis, 2008; Muckli, 2010; Boynton, 2011; Hillman, 2014). In particular, CBF and BOLD responses show better correlation with post-synaptic local field potentials (LFPs) than with spiking activity (multi-unit activity, MUA), suggesting that the hemodynamic response reflects stronger the input to a neuronal population in a brain area and intrinsic processing (Lauritzen, 2005) rather than the output of that area (Goense and Logothetis, 2008; Logothetis et al., 2010; Magri et al., 2012). Positive CBF and BOLD responses during stimulation are associated with an increase in neuronal activity and decrease in deoxyhemoglobin content, whereas negative CBF and BOLD responses are associated with a decrease in neuronal activity below baseline and increase in deoxyhemoglobin content (e.g., Shmuel et al., 2006 and references therein).

Typical positive BOLD responses to sustained stimulation display transients, such as response adaptation (also referred to as early-overshoot) and post-stimulus undershoot (Frahm et al., 1996; Krüger et al., 1996; Hoge et al., 1999). Similarly, neuronal responses to stimulation exhibit rapid rise followed by a decay (or adaptation) to a steady-state level and are often followed by a brief decrease below baseline after stimulus cessation (e.g., Logothetis et al., 2001). These neuronal transients (Boynton et al., 1996; Hoge et al., 1999; Bandettini and Ungerleider, 2001; Birn et al., 2001) are the result of changes in excitatory and inhibitory (E-I) balance between active neuronal populations, controlled by local micro-circuitry but also by long-range connections with other brain areas (Logothetis, 2002; Logothetis and Wandell, 2004; Shmuel et al., 2006; Hyder et al., 2010; Havlicek et al., 2017b). CBF reflects these neuronal transients in a temporally smoothed fashion (Hoge et al., 1999; Attwell and Iadecola, 2002; Uludağ et al., 2004; Sadaghiani et al., 2009; Attwell et al., 2010; Cauli and Hamel, 2010; Mayhew et al., 2014). Because of the complex underlying physiological processes, the BOLD response can exhibit transients not only from neuronal sources, but also due to the properties of blood vessels: the BOLD response is dominated by signal contributions originating from the venous compartments, and venous CBV can be dynamically uncoupled from CBF (i.e., venous CBV lags behind CBF), influencing the amplitude of the early-overshoot and post-stimulus undershoot of the BOLD-response (Buxton et al., 1998b; Mandeville et al., 1999; Yacoub et al., 2006; Chen and Pike, 2009). Alternatively, dynamic uncoupling between CBF and CMRO_2_ (i.e., CMRO_2_ lags behind CBF) could result in the same BOLD transients as well (Lu et al., 2004; Frahm et al., 2008; Donahue et al., 2009; Hua et al., 2011; Poser et al., 2011; van Zijl et al., 2012). However, we have recently argued, supported by modeling of experimental data, that the contribution of CBF-CMRO_2_ uncoupling to BOLD signal transients is much lower than that of CBF-venous CBV uncoupling (Havlicek et al., 2017a). Thus, neuronal and hemodynamic responses in different areas (or voxels) and subjects exhibit dynamic features, which can be both due to changes in E-I balance or due to biomechanical properties of the vasculature.

In standard analysis of fMRI data, linear convolution is applied between a stick or box-car functions (representing the stimulation paradigm) and assumed canonical hemodynamic response function (representing the combined transform from stimulus time-course to neuronal signal and the measured BOLD response) (Friston et al., 1995). However, as indicated above, excitatory and inhibitory neuronal responses may be nonlinearly related to stimulation (e.g., see Boynton et al., 1996; Bandettini and Ungerleider, 2001; Birn et al., 2001; Grill-Spector and Malach, 2001; Kida and Yamamoto, 2008; Mullinger et al., 2013, 2014; Pérez-González and Malmierca, 2014; Havlicek et al., 2017a; Keller et al., 2017). Furthermore, vascular transients resulting from dynamic uncoupling induce additional nonlinearities between the input function and subsequent hemodynamic variables. Therefore, the linearity assumption in BOLD data analysis may not be sufficient in many experiments and, consequently, the inferred information about the neuronal activity changes obtained from hemodynamic signals using linear (de)convolution analysis might be confounded with vascular effects. In other words, to more accurately estimate neuronal responses from the BOLD signal, a nonlinear model that accounts for dynamic relationships between neuronal and hemodynamic physiological variables underlying the BOLD response is needed.

Recently, we have introduced a physiologically-informed generative model of the BOLD signal within the framework of dynamic causal modeling (Friston et al., 2003) (called P-DCM)^1^, linking excitatory and inhibitory neuronal activity to the BOLD response (Havlicek et al., 2015). In P-DCM, we employ: (i) an adaptive excitation-inhibition neuronal model that accounts for a wide range of neuronal time-course both during stimulation and post-stimulation periods; (ii) a neurovascular coupling (NVC) model that links neuronal activity to blood flow in a strictly feedforward fashion; (iii) a balloon model (Buxton et al., 1998b) that can account for a vascular uncoupling between CBF and venous CBV; and (iv) field strength and sequence dependent parameterization of the BOLD signal equation. We compared P-DCM with other DCM models (Friston et al., 2003; Marreiros et al., 2008) and demonstrated significant improvements in the ability to model commonly observed neuronal and vascular response transients in single regions (Havlicek et al., 2015) and also within a network of several regions with task-driven activity changes (Havlicek et al., 2017b). In the latter case, we also showed a high fidelity of P-DCM to jointly explain CBF and BOLD responses simultaneously measured with the arterial spin labeling (ASL) fMRI technique, demonstrating the benefit of additional information provided by a CBF measurement for model inversion.

In general, multi-modal imaging is a powerful approach to study the relationship between neural activity and the BOLD fMRI signal. Measurements of different physiological variables can increase the ability to disambiguate neuronal and vascular effects present in the BOLD signal and potentially unravel limitations of the hemodynamic models. In the current work, we aim to explore the versatility of P-DCM to explain dynamic relationships between various combinations of measured physiological variables and to deduce the excitatory and inhibitory neuronal dynamics from hemodynamic data. This is done under the constraints of assumed physiological mechanisms and experimental manipulations. In particular, we use: (1) newly acquired CBF and BOLD responses to static and flickering stimuli in human subjects; (2) CBF, total CBV and BOLD response to square-wave grating stimulus acquired in the cat brain from the study of Jin and Kim (2008); and (3) positive and negative neuronal and BOLD responses induced by rotating visual stimuli measured in the monkey brain from the study of Shmuel et al. (2006). In our modeling, we emphasize stimulus-type-dependent modulation of response transients that can be linked to a dynamic interplay between excitatory and inhibitory activity. In addition, we allow for differences between stimulation and post-stimulation response periods and account for vascular-, magnetic field strength-, and MRI sequence-dependent properties. The current approach can also be generalized to other invasive and non-invasive multi-modal data, such as EEG-fMRI, provided generative models exist for both modalities.

## Methods

### General description of P-DCM

**Figure 1:**
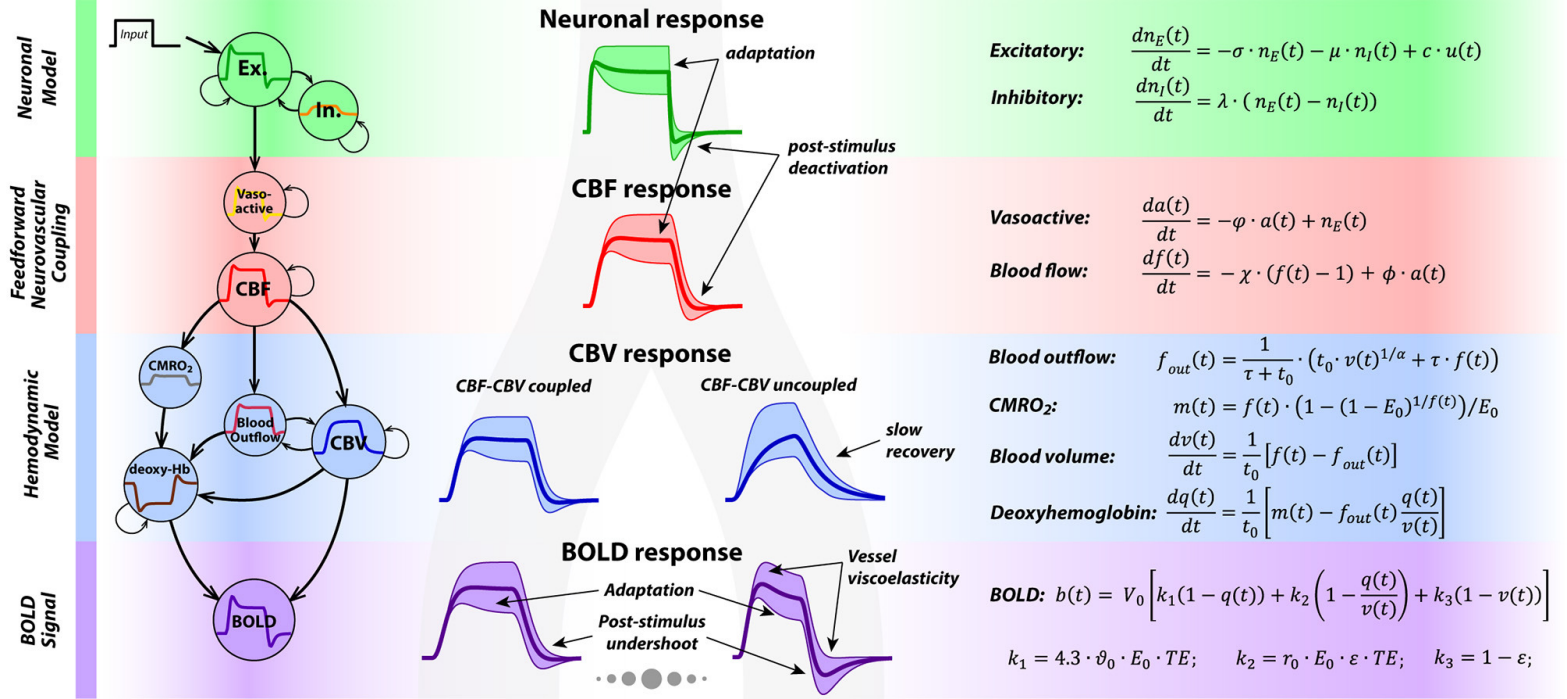
On the left, a schematic depicts the four main components of P-DCM generative model, representing the causal chain between the neuronal and the BOLD response. In the middle, shapes of the physiological responses generated at different stages of the generative models are shown. The shaded areas around the responses represent the amount of response transient variability that can be modeled by P-DCM. This illustration highlights the two main sources of BOLD response transients: caused either by (1) neuronal transients generated by the model of E-I balance; or by (2) vascular transients due to dynamic uncoupling between CBF and venous CBV; or mixture of these two. On the right, a summary of all equations underlying P-DCM is provided.

The generative model in P-DCM consists of four causally-linked components that define how the neuronal signal is transformed to the measured BOLD response (see Figure 1 for an illustration and the summary of model equations). For a more detailed description of the model and its comparison with previous DCM models, please see (Havlicek et al., 2015, 2017b).

#### Neuronal model of E-I balance

In this model, an exogenous input *u*(*t*) (e.g., sensory stimulus) drives the change in excitatory activity, *n*_*e*_(*t*), which is directly coupled with a change in inhibitory activity, *n*_*i*_(*t*). The strength of this input, expressed with the parameter *c*, scales the amplitude of the neuronal response. The shape of the neuronal response is tuned by the transient imbalance between excitatory and inhibitory activity. In particular, the typical overshoot at the onset can be produced by gradually increasing inhibitory activity that modulates the excitatory activity via negative feedback. Next, persistence of the inhibitory activity following stimulus cessation can produce post-stimulus deactivation. This temporal evolution of excitatory and inhibitory activity, including their dynamic mismatch, is controlled by the parameters σ and λ, respectively, and the strength of the inhibitory activity modulating the excitatory activity is encoded by the parameter μ. Optimization of these neuronal parameters allows modeling a broad repertoire of neuronal response adaptation profiles and of possible post-stimulus deactivations separately for the stimulation period (SP) and the post-stimulation period (PSP)^2^.

#### Neurovascular coupling (NVC):

The output of the neuronal model, i.e., the excitatory activity modulated by inhibitory activity, is transformed to CBF, *f*(*t*), in a strictly feedforward fashion, via vasoactive signal *a*(*t*). Neuronal excitation/inhibition leads to arterial vasodilatation/vasoconstriction associated with increased/decreased CBF (Devor et al., 2007). Thus, the modeled neuronal response transients are conveyed to a CBF response, albeit in a smooth version. Decay and delay of the CBF response with respect to the neuronal response is regulated with three constants, φ, ϕ and χ, with only χ being optimized during model inversion.

#### Hemodynamic model:

The hemodynamic model is represented by the balloon model (Buxton et al., 1998a,b). It models the mass balance of normalized changes in CBV, *v*(*t*), and deoxyhemoglobin content, *q*(*t*), as they pass through the venous compartment. Their changes are driven by changes in the inflowing CBF, *f*(*t*), and CMRO_2_, *m*(*t*), respectively. It is assumed that CBF and oxygen extraction faction, *E*(*t*), are dynamically coupled, thus $m\left(t\right)=f\left(t\right)\cdot \frac{E\left(t\right)}{E_{0}}$, where *E*_0_ is the value of oxygen extraction faction at rest (please see discussion of this assumption in (Havlicek et al., 2015), and the Discussion section for more details). Furthermore, during steady-state, the blood leaving the venous compartment (i.e., the outflow, *f*_*out*_(*t*)) and the venous CBV are coupled via a power law relationship (Grubb et al., 1974), with exponent α, whereas during the transient periods venous CBV and CBF can be uncoupled; i.e., venous CBV can evolve more slowly than CBF. This is due to the vessel's resistance to changes in venous CBV, described by the viscoelastic time constant, τ. Theoretically, the viscoelastic time constant can have different values during SP and PSP, τ_*SP*_ and τ_*PSP*_. The time dimension of changes in *v*(*t*) and *q*(*t*) is scaled by the mean transit time of the blood through the venous compartment at rest, *t*_0_. Note that *t*_0_ is linked to the resting venous blood volume fraction *V*_0_ via the central volume principle, $t_{0}=\frac{V_{0}\;}{F_{0}}$, where *F*_0_ is the blood flow at rest.

#### BOLD signal equation:

The BOLD signal reflects changes in the deoxyhemoglobin content, *q*(*t*), together with changes in the deoxyhemoglobin concentration, *q*(*t*)/*v*(*t*), and venous CBV, *v*(*t*). Their relative contribution is weighted by parameters that are magnetic field strength-, TE- and MRI sequence-dependent (Uludağ et al., 2009; Havlicek et al., 2015, 2017a).

In summary, as illustrated in Figure 1, P-DCM and its parameters allows the BOLD response to exhibit transients, such as response adaptation during stimulation and post-stimulus undershoot that can have both neuronal and vascular origins. As we show below, physiological origins of these transients can be tested under the constraints of concurrent multi-modal physiological data and experimental manipulations.

### Data description

To demonstrate the utility and versatility of P-DCM, below we describe three different data-sets acquired from human, cat, and monkey brains. Each data-set consists of a different combination of physiological measurements: I. CBF and BOLD response; II. CBF, total volume CBV, and BOLD responses, published in Jin and Kim (2008); and III. neuronal and BOLD responses, published in Shmuel et al. (2006). Any additional physiological data, next to the BOLD data (and/or experimental manipulation), provide physiologically-informed constraints on the underlying mechanisms of the BOLD response. This can result in a more accurate inference on the changes in E-I balance forming the neuronal responses (Havlicek et al., 2017b) and potentially inform about the limitations of model structure and parameter assumptions.

#### I. CBF and BOLD responses

Four healthy volunteers (females, age range: 26–34) were scanned for the current study on a 3 T Siemens Prisma^Fit^ MR scanner (Siemens Medical Solutions, Erlangen, Germany). For each subject, six functional runs and an anatomical scan were acquired. To obtain functional measurements of both CBF and BOLD signals, a multi-TE FAIR-Q2TIPS ASL sequence (Kim, 1995) was used with a gradient-echo echo-planar imaging (GE-EPI) readout and the following imaging parameters: TR = 2,200 ms; TI1/TI2 = 700/1,660 ms; TE1/TE2/TE3 = 8/21/33 ms; FOV = 192 × 192 mm^2^; nominal voxel size = 3 × 3 × 3 mm^3^, flip angle = 90°; matrix size = 64 × 64; 325 volumes (total scan duration 715 s); 10 oblique slices acquired in interleaved fashion, covering early visual areas. The anatomical MPRAGE scan was acquired with: 1 mm isotropic nominal voxel size; FOV = 224 × 224 mm^2^; matrix size = 224 × 224; TE = 2.1 ms; TR = 2,400 ms; TI = 1,040 ms.

The subjects were instructed to fixate on a small dot at the center of the screen throughout the experiments. Each of the EPI functional runs began with a 55 s resting period and continued with alternation of two static and two flickering checkerboard conditions (each 55 s long), interspersed with 110 s resting periods. Flickering checkerboards were presented at 4 Hz (eight reversals per second). The order of static and flickering conditions within a run was pseudo-randomized. For the static condition, a full-field, black-and-white radial checkerboard was presented (Michelson contrast 1), whereas, for the flickering condition, reduced contrast (Michelson contrast 1/3) checkerboards were presented at 4 Hz (i.e., 8 reversal per second) (Sadaghiani et al., 2009). The resting periods consisted of a gray screen isoluminant with the mean luminance of the checkerboard. In order to maintain the subjects' attention, the color change of the fixation dot (altering between red and blue at three pseudo-random intervals within each stimulation block) was passively observed. For each subject, there were twelve trials per condition.

For each subject, the CBF time-series were derived from the ASL data acquired with TE = 8 ms using surround subtraction (Mumford et al., 2006). The BOLD time-series were obtained from the ASL data acquired with TE = 33 ms using surround averaging. The data were preprocessed using SPM12 (R6470) (http://www.fil.ion.ucl.ac.uk/spm). To correct for head motion, the realignment parameters with respect to the first volume were estimated using BOLD data and the same realignment parameters were applied to the corresponding volumes of CBF data. The mean BOLD image was coregistered to the anatomical image and the estimated spatial transformation matrix was applied to the functional BOLD and CBF data. CBF data were modeled voxel-wise using a general linear model (GLM). This model included main predictors representing the periods of static and flickering visual stimulation and additional predictors representing the stimulus onset and offset. All predictors (i.e., three per condition) were convolved with a gamma-variate hemodynamic response function (“*spm_Gpdf.m*”) with shape and scale parameters 4 and 0.5, respectively. The additional predictors were introduced to explain deviations of the hemodynamic response shape between conditions during stimulation and post-stimulation periods. The predictors were not orthogonalized in order to retain a direct interpretation of the model. Furthermore, data were high-pass filtered (cut-off = 1/256 s) to remove low frequency signal drifts and a first-order autoregressive model was used to remove serial correlations. Based on a conjunction analysis of the two main contrasts for static and flickering conditions, significant voxels within the gray matter of the left and right visual cortex (*p* < 0.05, corrected for family-wise errors) were selected from the CBF data. The same voxels were selected from the BOLD data. The statistically significant BOLD signal map (not reported) included the CBF ROI, but had a larger spatial spread. Voxel's time-courses from the CBF and BOLD data (~40 voxels per subject) were extracted, high-passed filtered (cut-off = 256 s), and the average responses in percent signal change were calculated for the two experimental conditions.

#### CBF, total CBV and BOLD responses

Hemodynamic responses of CBF, total CBV, and BOLD signal were extracted from Figure 1 of the paper by Jin and Kim (2008) using Matlab (MathWorks, Inc.). These responses were measured using fMRI in the visual cortex of anesthetized cats at 9.4 T. In brief, they used 60 s visual stimulus of black and white square-wave gratings drifting with a temporal frequency of 2 cycles/s. This was always preceded by 20 s and followed by 60 s control condition represented by the same but stationary gratings. All data were acquired with GE-EPI readout. BOLD and total CBV signals were acquired simultaneously with TR = 2 s and ~0.2 × 0.2 × 2 mm^3^ voxel size and CBF signal was acquired during separate runs with TR = 3 s and ~0.3 × 0.3 × 2 mm^3^ voxel size. However, the reported responses from Figure 1 were upsampled (using linear interpolation) to TR = 1 s. BOLD responses were derived from two TEs of 10 ms and 20 ms (by calculating the slope $\Delta R_{2}^{*}$, but displayed in average percent signal change). Furthermore, in Figure 1 by Jin and Kim, hemodynamic responses were reported for both the middle and top (superficial) part of the gray matter. We have taken CBF and total CBV responses from the middle gray matter and the BOLD response from the superficial area, where it exhibited the highest signal change. Note that CBF and total CBV are more localized to signal changes induced in the arterial blood compartment, while BOLD signal is mostly represented by the venous compartment, where the draining veins carry the signal from deeper gray matter structures toward the surface. Therefore, from the view of blood dynamics, the BOLD response measured at the surface mostly reflects the CBF and arterial CBV changes that occurred deeper in the gray matter. Finally, for all three hemodynamic responses, it is important to preserve the exact amounts of reported percent signal changes.

#### III. Neuronal and BOLD responses

**Figure 2:**
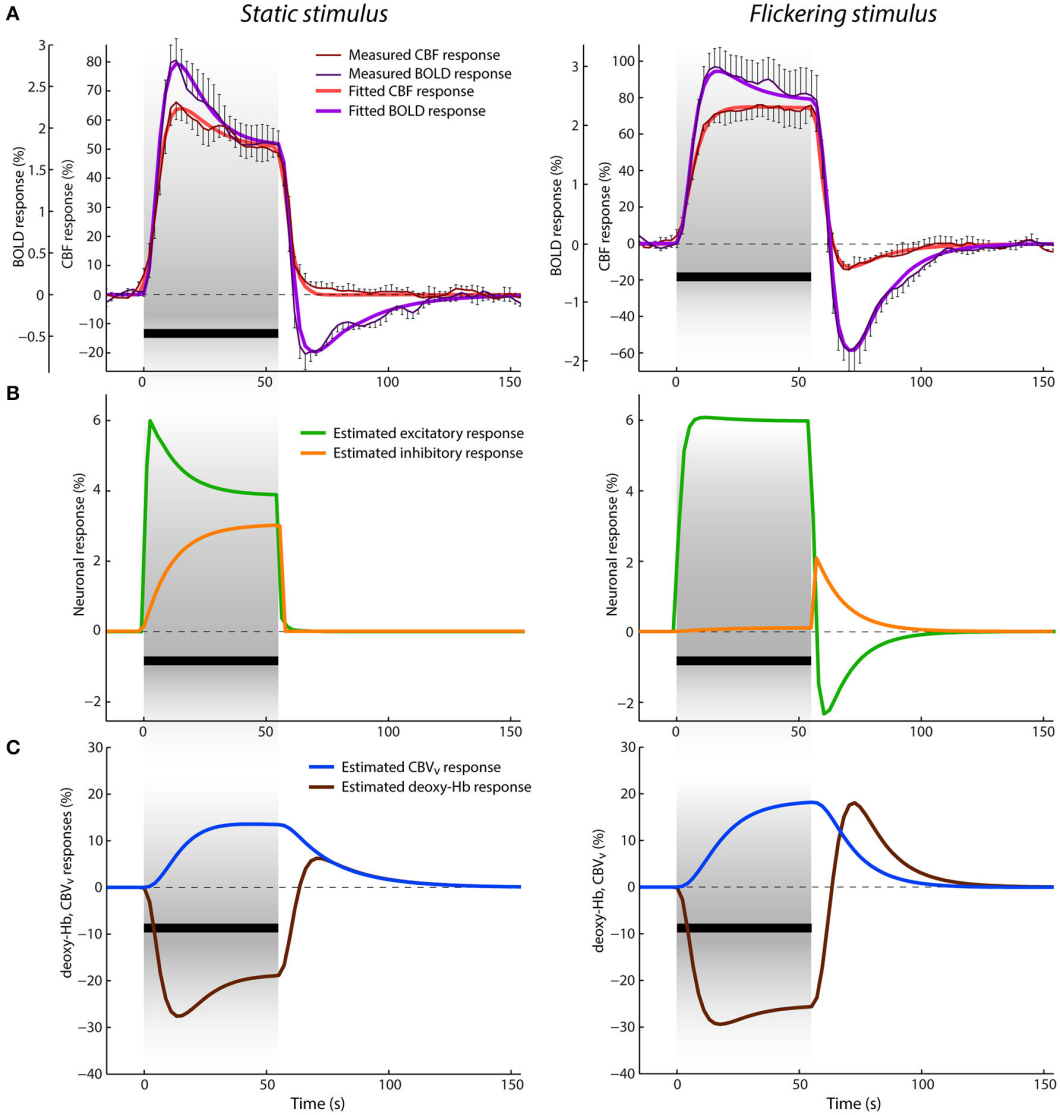
Data and results depiction of experiment I. **(A)** The average CBF and BOLD responses derived from the ASL data for static (left) and flickering (right) conditions displayed in percent signal change with thin dark red and purple lines, respectively. The error-bars represents the standard error of the measurement (*n* = 5). The measured responses are overlaid with the fitted CBF and BOLD responses, displayed with thick red and purple lines. The black bar below the responses represents the stimulation period. **(B)** Estimated excitatory (green lines) and inhibitory (orange lines) responses in percent signal change. **(C)** Estimated venous CBV (blue lines) and deoxyhemoglobin content (brown lines) responses in percent signal change.

Neuronal and BOLD responses were extracted from Figures 2A, 1D of the paper by Shmuel et al. (2006), respectively. These responses were measured simultaneously using invasive electrophysiological recording and fMRI in the visual cortex of anesthetized monkey brain at 4.7 T. In brief, 20 s visual stimuli consisting of high-contrast radial checkers rotating 60° per s were presented on gray background. The same background was used 5 s before and 25 s after the stimuli. Stimulation ring overlapping with the receptive field at V1 induced positive response in the vicinity of the electrode, while stimulus ring, which did not overlap with the receptive field, induced negative response in the same area. Neuronal responses were obtained by averaging the fractional change in power spectrum over the whole range of frequencies (4–3,000 Hz) with temporal resolution of 1 s. BOLD data were acquired with GE-EPI readout, TE = 20 ms, TR = 1 s and in-plane spatial resolution of 0.75 × 0.75 × 2 mm^3^. Positive and negative BOLD responses induced by two stimuli were sampled and averaged over the same voxels within the ROI around the electrode.

### Model specification

The multi-modal data from each study is used to identify the neuronal and vascular parameters of the generative model. Below, we specify the model assumptions for each study and form the observation equation to enable joint fitting to multiple physiological variables. In general, we aim to constrain the model estimation by at least two physiological measurements, experimental manipulations and properties of the venous blood compartment, the latter being independent of experimental manipulations, as it is given by the biomechanical properties of blood vessels. The assumptions about time-period- (i.e., stimulation or post-stimulation period) and experimental condition-specificity of certain model parameters were motivated by three criteria: (1) we favor the minimum number of parameters that can sufficiently explain the dynamic behavior of the multi-modal experimental data; (2) we have prior knowledge from previous results that some parameters have to be time-period- and/or experimental condition-specific, e.g., vascular parameters are condition independent for the same voxels; (3) the first and second criteria should be consistent for all three experiments described above, i.e., the same assumptions about neuronal and vascular parameters have to hold for all three experiments. Furthermore, if available, the model is also constrained by the measured percent signal changes of CBF and BOLD responses.

#### Experiment I: CBF and BOLD responses:

To jointly model CBF and BOLD responses using P-DCM during both static and flickering conditions, we made the following specifications for the generative model in order to determine the transfer of condition-dependent neuronal changes to changes in the measured signals. Two independent inputs, *u*_*S*_ and *u*_*F*_, in the form of box-car functions representing 55 s long static and flickering visual stimulation (whose strengths were controlled by two parameters *c*), were used to drive the neuronal activity. To accommodate the assumption that each type of visual stimulus can result in a different adaptation profile during the stimulation period (SP) but also exhibit differences in neuronal adaptation during the post-stimulus period (PSP), parameters of the neuronal model, σ and μ, were allowed to vary between the two phases but also between conditions. On the other hand, λ was allowed to vary only between the two conditions. This is because after the static stimulus the CBF response exhibits a slower return to the baseline without a post-stimulus undershoot, which is effectively modeled by setting the parameter μ close to zero. Thus, during this PSP, parameter λ does not have an effect on the shape of the neuronal response and becomes unidentifiable. Further, the NVC parameter χ, was assumed to be the same for both conditions and SP and PSP. Within the hemodynamic model, the viscoelastic time constant, τ, controlling the expansion and deflation of the venous compartment was allowed to vary between SP and PSP but not during the two conditions. The mean transit time at rest, *t*_0_, was estimated together with the resting blood volume fraction, *V*_0_, by assuming a blood flow value at rest, *F*_0_ = 0.01 s^−1^ (i.e., 60 ml/100 g/min, a typical value for human visual cortex; Donahue et al., 2006). All these free parameters and their usage during specific periods and conditions are summarized in Table 1. Next, the Grubb's exponent α and the oxygen extraction fraction at rest, *E*_0_, were fixed to 0.3 and 0.35, respectively. The BOLD signal equation was parameterized for 3 T magnetic field strength and the sequence parameters utilized in this study (see Table S1).

**Table 1:** Model parameters optimized during model inversion of the three data-sets.

		**Free parameters**						
		**Neuronal model**				**NVC**	**Hemodynamic model**	
		***c* (-)**	**σ (s^−1^)**	**μ (s^−1^)**	**λ (s^−1^)**	**χ (s^−1^)**	**τ (s)**	***t*_0_ (s), *V*_0_ (%)**
**CBF AND BOLD DATA:**								
SP	Static	■	■	■	■^†^	■	■	■
	Flicker	■	■	■	■^‡^			
PSP	Static	-	■	■	^†^		■	
	Flicker	-	■	■	^‡^			
**CBF AND TOTAL CBV AND BOLD DATA:**								
SP	Gratings	■	■	■	■	■	■	■
PSP		-	■	■				
**NEURONAL AND BOLD DATA:**								
SP	Positive	■	■	■	■^†^	■^†^	■	■
	Negative	■	■	■	■^‡^	■^‡^		
PSP	Positive	-	■	■	^†^	^†^	■	
	Negative	-	■	■	^‡^	^‡^		

*■Indicates parameter that is optimized within a certain time-period or condition*.

†‡ *Indicates optimized parameters that were consider the same between time-periods but different between conditions (e.g., †marks the static condition and ‡marks the flickering condition in the first experiment)*.

The modeled physiological variables were linked to measured (averaged) response at the level of CBF (i.e., the output of NVC) and BOLD signals. To enable their joint fitting, we considered a concatenated form of the observation equation:

(1) $$\left[\begin{array}{c} y_{f}\\ y_{b} \end{array}\right]=\left[\begin{array}{c} f-1\\ b \end{array}\right]\;\cdot \;100+\left[\begin{array}{c} \varepsilon_{f}\\ \varepsilon_{b} \end{array}\right],$$

where *y*_*f*_ and *y*_*b*_ are measured CBF and BOLD responses to both static and flickering stimuli in percent signal changes, all concatenated to a single vector. The measured data were explained by modeled CBF and BOLD responses, *f* and *b*, respectively, with additive error terms ε_*f*_ and ε_*b*_, constituting the “AR(1)+white noise” model (Friston et al., 2003).

#### Experiment II: CBF, total CBV and BOLD responses:

To jointly model CBF, total CBV and BOLD responses to the same visual stimulus, the generative model was specified as follows: the excitatory activity of the neuronal model was driven by a single input in the form of a box-car function representing 60 s stimulation duration, scaled by the parameter *c*. As in the previous experiment, the neuronal parameters σ and μ were allowed to vary between SP and PSP periods, while a single λ was estimated for both periods. The NVC and hemodynamic model were also controlled in the same way as in the previous experiment (see Table 1). The BOLD signal equation was parameterized for 9.4 T magnetic field strength and specific sequence parameters (see Table S1). Since, we have additional measurements of the total CBV, the observation equation had the following form:

(2) $$\left[\begin{array}{c} y_{f}\\ y_{\tilde{v}}\\ y_{b} \end{array}\right]=\left[\begin{array}{c} f-1\\ \tilde{v}\\ b \end{array}\right]\;\cdot \;100+\left[\begin{array}{c} \varepsilon_{f}\\ \varepsilon_{\tilde{v}}\\ \varepsilon_{b} \end{array}\right].$$

Here, the total CBV data, *y*_ṽ_, is modeled as a weighted sum of CBF (i.e., approximating the response shape of arterial CBV) and venous CBV, ṽ = *w*_*a*_ · (*f* − 1) + *w*_*v*_ · (*v* − 1), with weights *w*_*a*_ and *w*_*v*_ scaling the contribution of arterial CBV (i.e., proportional to CBF) and venous CBV. In other words, the measured BOLD data constraints the relative contributions of arterial and venous CBV to the measured total CBV data, as in the BOLD signal model only the venous CBV contributes to its time-course.

### Experiment III: neuronal and BOLD responses:

To jointly model positive and negative neuronal and BOLD responses to non-overlapping and overlapping visual stimuli, respectively, the generative model was specified as follows: Two independent inputs, *u*_*P*_ and *u*_*N*_, scaled by the input strength parameters were used to induce positive and negative responses, respectively. The stimulus duration for the positive response was 25 s but 26 s for negative response as the measured neuronal response remained decreased ~1 s after stimulus cessation. Furthermore, in contrast to the two experiments above, we assumed that NVC can differ for the positive and negative responses (i.e., a response-type-specific χ), as the mechanisms for NVC may differ for increases and decreases in CBF. On the other hand, similarly as before, the dynamic properties of venous blood compartment (i.e., the viscoelastic time constant τ) were assumed the same for the two response types but possibly different between SP and PSP periods. Thus, also a single mean transit time, *t*_0_, and resting venous blood volume, *V*_0_, were assumed across both conditions (see Table 1). The BOLD signal equation was parameterized for 4.7 T magnetic field strength and specific sequence parameters (see Table S1). Since in this experiment we also have access to neuronal recordings, the observation equation had the following form:

(3) $$\left[\begin{array}{c} y_{n}\\ y_{b} \end{array}\right]=\left[\begin{array}{c} \begin{array}{c} w_{n}\;\cdot \;n_{e} \end{array}\\ b \end{array}\right]\;\cdot \;100+\left[\begin{array}{c} \varepsilon_{n}\\ \varepsilon_{b} \end{array}\right],$$

where *y*_*n*_ and *y*_*b*_ are the measured neuronal and BOLD responses to both types of stimuli (i.e., both positive and negative responses, concatenated to a single vector) in percent signal changes. Since the reported percent signal change of the measured neuronal responses in Shmuel et al. (2006) do not directly relate to the physiologically plausible range of CBF and BOLD signal changes in our model, the excitatory neuronal response, *n*_*e*_, in the observation equation was additionally scaled with the parameter *w*_*n*_.

### Model inversion

Modeled responses were calculated by a numerical integration of differential equations using a local linearization approach (Ozaki, 1992), with integration step Δ*t* = 0.1 s and later downsampled to match the TR of the measured data. Responses defined by the above observation equations were fitted to the measured data using variational Laplace (VL) optimization algorithm (Friston et al., 2007) as implemented in the SPM12 toolbox (“*spm_nlsi_GN.m*”). This is a Bayesian estimation procedure designed for the estimation of nonlinear dynamic models, where the model parameters are specified in terms of priors. It calculates posterior parameter estimates by iteratively maximizing the free energy (i.e., the approximation to the model log-evidence). Since the VL algorithm employs the Laplace assumption, all the parameters—prior and posterior—are defined using Gaussian distributions. As most of the physiological parameters included in the model can only have positive values, thus, their default values are scaled with a latent variable via the log-normal transformations; e.g., $\tau \cdot \exp\left(\tilde{\tau }\right)$ (see Table S2). The prior means and variances of the latent variables are listed in Table S1.

## Results

### CBF and BOLD responses

Figure 2A shows the average CBF and BOLD responses to static and flickering stimuli in percent signal changes, respectively. With the onset of the static stimulus, the CBF response rises first and then the BOLD response follows slightly later. They both reach their maxima (i.e., 66% for CBF and 2.8% for BOLD) after ~13 s and continue with a steady decrease toward the end of stimulation. This response adaptation has more pronounced character in the BOLD response. After stimulus offset, both responses rapidly decrease. The amplitude of the CBF response first drops quickly to ~10% and then slowly recovers to the baseline. In contrast, the amplitude of the BOLD response drops below baseline, with a negative peak of −0.7%, at ~11 s after stimulus offset. This post-stimulus undershoot then recovers to the baseline in the next ~60 s. The CBF and BOLD responses to the flickering stimulus differ substantially from the responses to the static stimulus. The CBF response reaches its maximum (i.e., 77%) only by the end of the stimulation, exhibiting mostly a flat plateau from ~22 to 55 s. In contrast, the BOLD response peaks to 3% during an earlier phase of the stimulation, i.e., after 15 s, which is slightly later compared to the BOLD response to the static stimulus. From this time point, the BOLD response slightly and slowly decreases toward the end of the stimulation. After stimulus cessation, both CBF and BOLD responses drop below baseline (reaching their negative peaks in ~15 s after stimulus offset, at −15 and −1.8%, respectively) and then slowly recover to baseline. In general, the post-stimulus BOLD undershoot is much larger and peaks slightly later compared to the post-stimulus undershoot in the CBF response (both relative to their respective positive responses). Additionally, the post-stimulus BOLD undershoot recovery to baseline is steeper compared to the CBF response.

The results of jointly fitting CBF and BOLD responses using the P-DCM model are overlaid on the measured data in the same Figure 2A. The estimated model parameters are listed in Table 2. One can see that the model was able to accurately explain the discrepancy in the response shape of the two hemodynamic variables, and also the response shape variation due to differences in the type of visual stimuli (see fitted CBF and BOLD responses depicted with thick red and purple lines, respectively).

**Table 2:** Estimated values of model parameters.

		**Free parameters**									
		**Neuronal model**				**NVC**	**Hemodynamic model**		**Additional**		
		***c* (-)**	**σ (s^−1^)**	**μ (s^−1^)**	**λ (s^−1^)**	**χ (s^−1^)**	**τ (s)**	***t*_0_ (s), *V*_0_ (%)**	***w*_*n*_ (-)**	***w*_*a*_ (-)**	***w*_*v*_(-)**
**EXPERIMENT I: CBF AND BOLD RESPONSES**											
SP	Static	0.08	1.15	0.73	0.05^†^	0.28	68.68	2.07	-	-	-
	Flicker	0.04	0.52	0.01	0.05^‡^						
PSP	Static	-	0.25	0.00	†		69.49				
	Flicker	-	0.66	0.39	‡						
**EXPERIMENT II: CBF AND TOTAL CBV AND BOLD RESPONSES**											
SP	Gratings	0.17	2.80	7.30	0.02	0.27	59.91	2.06	-	0.12	0.19
PSP		-	2.13	1.95			41.11				
**EXPERIMENT III: NEURONAL AND BOLD RESPONSES**											
SP	Positive	0.13	1.87	3.19	0.15^†^	0.19^†^	9.37	2.95	12.69^†^	-	-
	Negative	−0.09	1.32	6.70	0.06^‡^	0.85^‡^			2.59^‡^		
PSP	Positive	-	1.89	1.94	^†^	^†^	28.53		^†^		
	Negative	-	1.05	1.70	^‡^	^‡^			^‡^		

†‡ *Indicates optimized parameters that were consider the same between time-periods but different between conditions as described in Table 1*.

The estimated excitatory and inhibitory neuronal responses are displayed in Figure 2B. The excitatory and not the inhibitory neuronal response mostly defines the shape of the CBF response. However, it evolves faster, with more pronounced transients, such as response adaptation in the case of the static condition and post-stimulus deactivation in the case of the flickering condition. Response adaptation and post-stimulus deactivation were explained by an amplitude variation of the inhibitory neuronal response. This means that the more pronounced response adaptation during stimulation is caused by a larger, gradual increase of inhibitory activity above baseline and the post-stimulus deactivation solely reflects the sharp increase of inhibitory activity after stimulus cessation (followed by a slower return to baseline). Note that the inhibitory responses displayed in Figure 2B were modulated by the period and condition specific parameter μ (i.e., the inhibitory-to-excitatory coupling). These experimental data and modeling results demonstrate that the type of stimulus modulates both the positive response and the post-stimulus undershoot, but in a different manner. This means that the E-I balance changes with time and can be very different between stimulation and post-stimulation periods, which is reflected in estimated neuronal parameters (i.e., in σ and μ).

The discrepancy between the measured CBF and BOLD responses was explained with uncoupling between the CBF and venous CBV responses. In Figure 2C, we can see that the venous CBV time-course evolves in a much slower fashion than the CBF time-course (Figure 2A). For example, for the static stimulus, the venous CBV response slowly increases during stimulation while the CBF response starts declining already after ~15 s of stimulation. Similarly, the CBF response returns much faster to baseline after stimulus cessation than the venous CBV response. This dynamic uncoupling during the transient periods results in a more pronounced response transients in the BOLD response, which approximately represents the inverted deoxyhemoglobin response (see Figures 2A,C). The CBF-venous CBV uncoupling parameterized by the viscoelastic time constant was estimated separately for the stimulation and post-stimulation periods, but yielded almost identical values τ_*SP*_ ≅ 68 *s* and τ_*PSP*_ ≅ 69 *s*, respectively. These large values reflect the fact that τ should scale with stimulus duration (see Uludağ and Blinder, 2017 and references therein). Importantly, the discrepancy between CBF and BOLD responses was explained with the same viscoelastic time constants for both the static and flickering conditions. This demonstrates that the passive mechanism of CBF-venous CBV uncoupling is independent of stimulus type (but dependent on the stimulus duration).

### CBF, total CBV and BOLD responses

**Figure 3:**
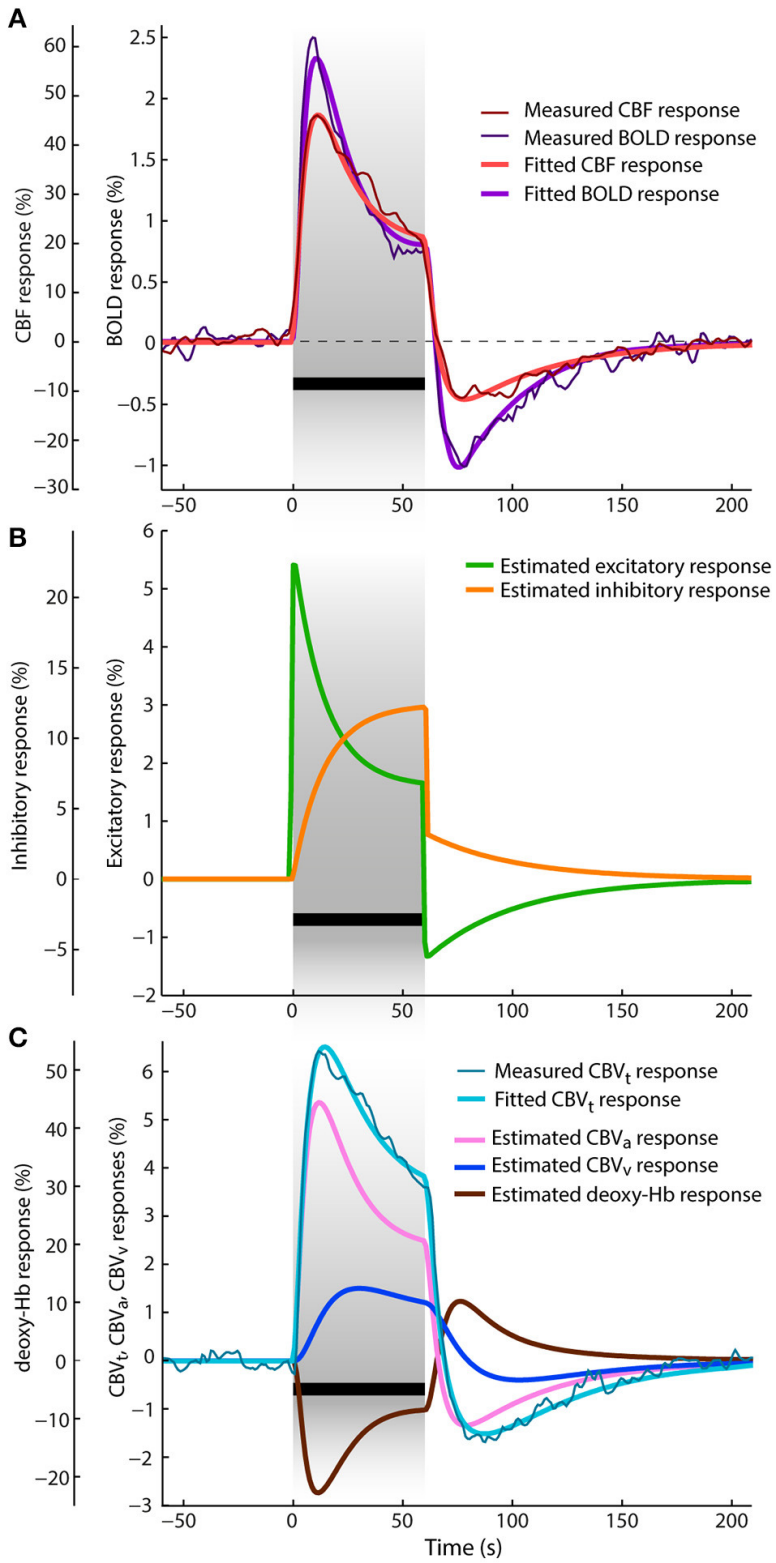
Data and results depiction of experiment II. This figure and its sections follow the same plotting format as Figure 2. The average CBF, BOLD and total CBV responses are replotted versions of the data reported in Figure 1 of Jin and Kim (2008). (A) The fitted and average measured CBF and BOLD responses. (B) The estimated excitatory and inhibitory responses. (C) The average measured and fitted total CBV responses are displayed with dark thin and light think cyan lines, respectively. Next, to the estimated venous CBV response and deoxyhemoglobin content responses, also the arterial CBV response is depicted (pink line).

Figure 3A shows the averaged CBF and BOLD responses in percent signal change to 60 s visual stimulation as reported by Jin and Kim (2008). The CBF response reaches its maximum peak (at 46%) ~12 s after the stimulus onset. The BOLD response reaches the peak ~2 s earlier (at 3.5%), even though the CBF response is faster immediately after stimulus onset. The average total CBV response (displayed in Figure 3C in percent signal change) rises with the BOLD response but its peak (at 6.5%) is ~3 s delayed with respect to the BOLD response peak. After reaching their maxima, all three responses decrease toward the end of stimulation. While the BOLD response exhibits the steepest decay, the decrease of the total CBV response is the slowest. The amplitudes of CBF, total CBV and BOLD at the end of stimulation are 21, 3.6, and 1% (i.e., in ratios of 0.46, 0.55, and 0.29 with respect to their maximum peaks), respectively. After stimulus cessation, all responses drop below baseline and exhibit considerable post-stimulus undershoots. The ratios of the post-stimulus response undershoots with respect to the amplitudes at the end of the stimulation for CBF, total CBV, and BOLD are 0.57, 0.42, and 1.4, respectively. The BOLD response with the largest relative post-stimulus undershoot reaches the negative peak earliest (at −1.4%), i.e., ~18 s after stimulus onset, then CBF with smaller relative undershoot follows (−12%, after 20 s), and the total CBV response has the smallest and most sluggish relative post-stimulus undershoot (−1.5%, after 27 s). All responses take almost 100 s to fully recover to baseline.

The results of jointly fitting CBF, total CBV and BOLD responses using the P-DCM model are overlaid on the measured data in the same Figures 3A,**C**. The estimated model parameters are listed in Table 2. All fitted CBF, total CBV and BOLD responses follow very closely the dynamic changes observed in the experimental data. The estimated excitatory neuronal response depicted in Figure 3B shows a strong and fast adaptation during stimulation and drops significantly below baseline immediately after stimulus cessation, followed by a slow recovery to baseline. As in the previous study, neuronal response adaptation during stimulation and post-stimulus deactivation are modeled by dynamic changes in the inhibitory neuronal response. Inhibitory response modulates the excitatory response by different amounts during stimulation and post-stimulation periods (see Table 2, for differences in optimized period-specific neuronal parameters).

**Figure 4:**
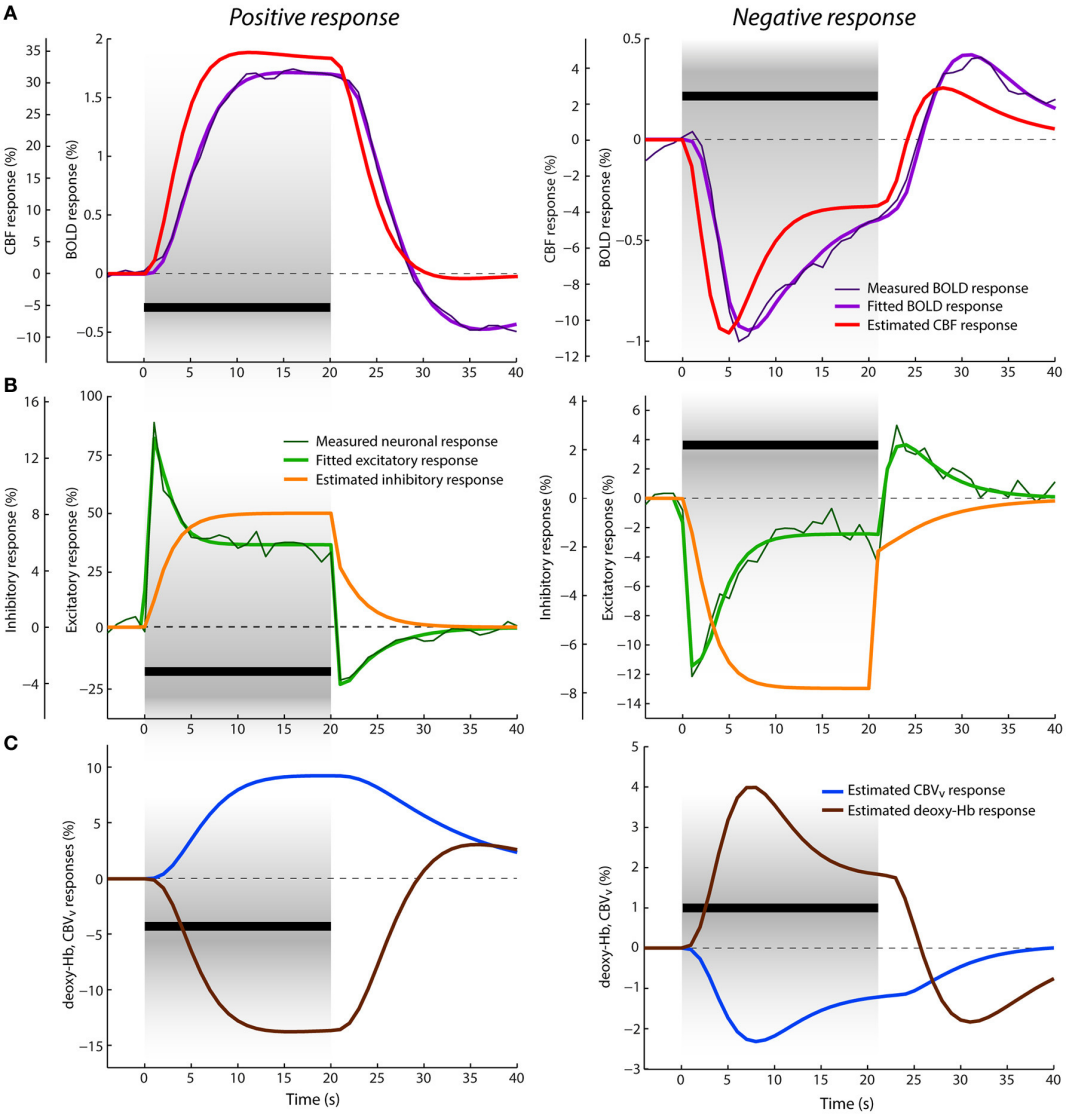
Data and results depiction of experiment III. This figure and its sections follow the same plotting format as Figure 2. The average positive (left) and negative (right) neuronal and BOLD responses are replotted versions of the data reported in Figures 1D, 2A of Shmuel et al. (2006). (A) The estimated CBF and fitted BOLD (and average measured BOLD) responses. (B) The average measured neuronal responses are depicted with dark thin green lines overlaid with fitted excitatory responses displayed with thick green lines. (C) The estimated venous CBV and deoxyhemoglobin content responses in percent signal change.

The dynamic relationship between CBF, total CBV, and BOLD signal was explained with the CBF-venous CBV uncoupling. Estimated arterial and venous CBV (CBV_a_ and CBV_v_) responses are displayed in Figure 3C in percent signal changes as they contribute to the predicted total CBV response (weighted by parameters *w*_*a*_ and *w*_*v*_). One can see that the arterial CBV change is larger than the venous CBV change (e.g., by a factor of ~2 at the end of the stimulation) and that venous CBV evolves slower during the transient periods. The large dynamic uncoupling between CBF (or arterial CBV) and venous CBV, which differed between stimulation and post-stimulations periods (τ_*SP*_ ≅ 60 s and τ_*PSP*_ ≅ 41 s), was estimated to significantly contribute to the size of BOLD response transients. These τ values provide a good explanation for the significantly more pronounced response adaptation and post-stimulus BOLD undershoot compared to the CBF and total CBV responses (as also reflected by the ratios mentioned above). As a consequence of CBF and arterial CBV responses exhibiting strong post-stimulus undershoots (due to the aforementioned decrease of excitatory activity below baseline during PSP), also venous CBV shows a post-stimulus undershoot, even though it is reduced and smoothed due to the viscoelastic properties of veins (i.e., large τ) (see Figure 4C). The same mechanism applies for the slower increase and strongly reduced adaptation profile of venous CBV during SP.

### Neuronal and BOLD responses

Average positive and negative neuronal responses in percent signal changes to 20 s visual stimuli overlapping and non-overlapping with its receptive field, respectively, as reported in Shmuel et al. (2006), are displayed in Figure 4B. The corresponding average positive and negative BOLD responses (also in percent signal changes) are displayed in Figure 4A. The positive neuronal response reaches its maximum peak immediately after stimulus onset (given downsampling of the neuronal signal to TR = 1 s). Then, within the next 5 s, it rapidly decreases to its lower plateau, where it remains till the end of the stimulation. In contrast, the positive BOLD response shows a rather slow increase, reaching its maximum ~15 s after stimulus onset (at 1.75%) and it remains about this level until the end of the stimulation. After stimulus cessation, the neuronal response drops quickly below baseline, producing a strong post-stimulus deactivation followed by gradual return to baseline in the next ~13 s. The BOLD response also decreases after stimulus cessation but in a much slower fashion, crossing the baseline ~9 s after the stimulus offset. Then it continues with a stronger post-stimulus BOLD undershoot, which is significantly delayed (~15 s) with respect to the neuronal post-stimulus deactivation. The limited post-stimulus period of 25 s did not allow for a full recovery of the BOLD undershoot to baseline. The negative neuronal response can be seen as an inverse of the positive response, but exhibiting significantly smaller signal change. After stimulus onset, there is an immediate decrease in neuronal activity below baseline followed by adaptation to the plateau of lower sustained amplitude during SP. After stimulus offset, it first slightly decreases^3^ (within 1 s) and then quickly increases, showing a post-stimulus activation above baseline, which is mostly in phase with the post-stimulus deactivation observed in the positive neuronal response. The negative BOLD response follows the course of the neuronal response more closely compared to the positive BOLD response. It reaches the minimum peak (at ~1%) ~6 s after the stimulus onset and keeps increasing almost linearly toward the end of the stimulation (to −0.4%). Afterwards, it crosses baseline ~4 s earlier than the positive BOLD and exhibits a post-stimulus BOLD overshoot (with maximum at 0.4%) that evolves significantly faster than the post-stimulus undershoot of the positive response.

The results of jointly fitting the positive and negative neuronal and BOLD responses with P-DCM are overlaid with the measured data in Figures 4B,A, respectively. The estimated model parameters are listed in Table 2. The fitted neuronal and BOLD responses follow very closely the dynamic changes observed in the experimental data. The estimated excitatory neuronal responses for both positive and negative responses provided an accurate representation of the transient features observed in the experimental data. As before, response adaption and post-stimulus deactivation profiles were modeled by a variable modulation of the excitatory activity by inhibitory activity (see Figure 4A). The dynamic features of the positive excitatory neuronal response are well comparable to the estimated neuronal response in the second experiment (or to the response features seen in the first experiment). The negative excitatory response was induced by the stimulus input function *u*_*N*_(*t*) scaled by the negative *c* parameter. Thus, in contrast to the positive neuronal response, the inhibitory response gradually decreases below baseline during SP, which then causes an increase of the excitatory activity after its initial drop. After stimulus cessation, the inhibitory activity quickly increases up to ~¼ of the total decrease, and then slowly recovers to the baseline. This slow post-stimulus recovery of the inhibitory response below baseline causes an increase (i.e., overshoot) in the post-stimulus excitatory response.

Next, as the NVC could differ between the positive and negative responses, the estimated positive CBF response is significantly delayed with respect to the neuronal response, which smooths out the strong adaptation during SP and post-stimulus deactivation observed in the neuronal response. This slow evolution of the CBF response was achieved by slowing down the feedforward mechanism of NVC (i.e., by lowering the decay constant χ). On the other hand, the estimated CBF response following the negative neuronal response is much faster, closely resembling dynamic features of the neuronal response. This is because the NVC acts faster (by employing a higher decay constant χ).

Furthermore, the fitted positive BOLD response is even more delayed with respect to the CBF response, with mean transit time at rest, *t*_0_ ≅ 3 s (see Figure 4B). The CBF-CBV uncoupling is smaller, with viscoelastic time constant, τ_*SP*_ ≅ 9 s, (see Figure 4C). Therefore, no response adaptation is present during SP. Although the estimated CBF response exhibits a minimal post-stimulus undershoot, the stronger post-stimulus BOLD undershoot is well explained by a larger CBF-venous CBV uncoupling (τ_*PSP*_ ≅ 29 s) during PSP. The venous CBV and deoxyhemoglobin responses are displayed in Figure 4C. The negative BOLD and CBF responses show similar response transients even though the same viscoelastic time constants, regulating CBF-venous CBV uncoupling during SP and PSP, were used as in the case of the positive response. Significant post-stimulus overshoot in the CBF response can account for a large fraction of the post-stimulus BOLD overshoot. This is because the actual effect of CBF-CBV uncoupling on the post-stimulus BOLD undershoot for the negative response is smaller due to a generally lower amplitude level of venous CBV during recovery (see Figure 4C). Therefore, in contrast to the positive response, the main origin of post-stimulus BOLD overshoot is neuronal.

## Discussion

The BOLD fMRI signal is an indirect reflection of neuronal activity. It has been suggested that it best correlates with the post-synaptic potentials, which—after mediation by metabolic and vascular processes—results in the characteristic hemodynamic delay and blurring relative to neuronal activity. Thus, the high complexity of tissue processes associated with brain activity, ranging from microscopic (i.e., molecular) to macroscopic (i.e., brain area) levels, is reduced to a spatially and temporally varying scalar number (i.e., the dynamic fMRI signal). That is, there is only reduced information about the excitatory and inhibitory neuronal activity available from the fMRI signal. As a result, temporal features of the BOLD signal, such as signal adaptation during stimulation or signal reduction after the stimulation, cannot be taken as a direct evidence of neuronal adaptation or post-stimulation deactivation, respectively. Recently, we have proposed, inspired by physiological observations, a novel generative hemodynamic model within the DCM framework, called P-DCM. We have demonstrated (using BOLD data and BOLD data combined with CBF) that P-DCM is superior in describing single ROI time-courses and also deducing the effective connectivity between brain areas (Havlicek et al., 2015, 2017b) compared to previous DCM models (Friston et al., 2003; Marreiros et al., 2008) and that the model inversion, in general, benefits from additional CBF data.

In this paper, we have additionally demonstrated the versatility of P-DCM to jointly explain dynamic relationships between neuronal, neurovascular and hemodynamic physiological variables underlying the BOLD signal using new and previously published multi-modal data. For this purpose, we utilized three data-sets of experimentally induced responses in primary visual areas measured in the brains of human, cat, and monkey, respectively: (1) CBF and BOLD responses to static and flickering stimuli acquired for this study; (2) CBF, total CBV and BOLD responses to square-wave grating stimulus (Jin and Kim, 2008); and (3) positive and negative neuronal and BOLD responses induced by overlapping and non-overlapping visual stimuli with the visual receptive field (Shmuel et al., 2006). The fitting of P-DCM to multi-modal data (i.e., the model inversion) was performed using a VB approach (Friston et al., 2007) under the constraint of assumed physiological mechanisms and experimental manipulations. Specifically, we assumed that the BOLD response transients, such as positive response adaptation and post-stimulus undershoot, can be due to two physiological mechanisms: (1) neuronal, due to changes in E-I balance caused by a dynamic interaction between excitatory and inhibitory neuronal populations (Hoge et al., 1999; Krekelberg et al., 2006; Shmuel et al., 2006; Logothetis, 2008; Sadaghiani et al., 2009; Mullinger et al., 2013); and (2) vascular, due to dynamic uncoupling between CBF and venous CBV responses (Mandeville et al., 1998; Chen and Pike, 2009; Kim and Ogawa, 2012; Huber et al., 2014a; Havlicek et al., 2017a). We also assumed that the experimental manipulation can modulate the neuronal response transients by changing the E-I balance and that this can differ between SP and PSP. Similarly, the vascular uncoupling was allowed to vary between SP and PSP but was invariant with respect to the experimental manipulations. P-DCM provided accurate fits to all measured multi-modal responses and was able to shed a light on the dynamic relationships between the physiological processes underlying the BOLD response. The limitations of P-DCM due to its assumptions are discussed below.

In the first experiment using both CBF and BOLD responses, we were able to show that a 55 s long static and flickering stimuli induced different modulations of the CBF response transients during SP and PSP and that there was a large discrepancy in the size and form of transients between the CBF and BOLD transients, as commonly observed (see e.g., Sadaghiani et al., 2009; Havlicek et al., 2017a and references therein). P-DCM could explain the experimentally induced modulation of the CBF response transient by optimizing the balance between excitatory and inhibitory activity. The accurate fit of both CBF responses to static and flickering stimuli was achieved by allowing some of the neuronal model parameters (σ, μ) to be time-period- and condition-specific, while others (λ) including the NVC parameter (χ) were considered condition- and period-invariant (see Table 2). Next, the large discrepancy in the size of transients between the measured CBF and BOLD responses was explained with a strong uncoupling between CBF and venous CBV responses, which was identified to be similar for SP and PSP (τ_*SP*_ ≅ 68 *s and τ*_*PSP*_ ≅ 69 *s*). More importantly, both BOLD responses to static and flickering stimuli could be explained by assuming the same vascular uncoupling for the two conditions. Additionally, these estimates compared quite well with our previous results obtained by applying P-DCM to the single-subject BOLD responses of the same experimental ASL data (Havlicek et al., 2015). Nevertheless, additional information about the shape of CBF responses incorporated in the current study provided more accurate estimation of the neuronal and vascular component contribution to the BOLD response transients (see Figure 6C in Havlicek et al., 2015).

By using P-DCM to explain the dynamic discrepancy in the shape of the CBF, total CBV and BOLD responses to a 60 s long square-wave grating visual stimulus provided by the second experiment (Jin and Kim, 2008), we were able to accurately jointly fit all measured responses. First, even though the response transients (i.e., early-overshoot and post-stimulus undershoot) were strongly present in both CBF and total CBV responses, the BOLD response transients were even more significantly pronounced (see Figure 3A). Thus, some additional mechanism next to the neuronal contribution is necessary to fully explain the BOLD response transients: As in the first study, the shape of CBF response was well explained by optimizing the E-I balance for the SP and PSP. Importantly, by having measurements of both CBF and total CBV responses underlying the BOLD response, we were able to determine that arterial CBV has a larger contribution to the total CBV than the venous CBV, which is in good agreement with other experimental observations (Drew et al., 2011; Kim and Kim, 2011; Huber et al., 2014a; Gagnon et al., 2015), and that venous CBV evolves much slower compared to the CBF (or arterial CBV) due to a strong (but slightly different) CBF-venous CBV uncoupling between SP and PSP (τ_*SP*_ ≅ 60 s and τ_*PSP*_ ≅ 41 s) (Uludağ and Blinder, 2017). The strong neuronal transients are well reflected in both the CBF and arterial CBV responses, and also the venous CBV response transients reflect this neuronal modulation, albeit largely smoothed out by the strong CBF-venous CBV uncoupling (see Figure 3C). Therefore, even though the venous CBV does not exhibit the more typical slow increase during SP and slow return to baseline during PSP (Kim and Kim, 2011; Huber et al., 2014a), the vascular uncoupling still significantly contributes, in addition to the CBF post-stimulus deactivation, to the post-stimulus BOLD undershoot (having ~50% neuronal and 50% vascular origin). Thus, our modeling results agree with a suggestion by Jin and Kim (2008) that there is a significant contribution of CBF post-stimulus deactivation to the post-stimulus BOLD undershoot, but disagree with their suggestion that these multi-modal data do not support the contribution of the vascular uncoupling between CBF and venous CBV. It is incorrect to assume that the venous CBV must exhibit slow return to baseline after stimulus cessation in order to effectively contribute to the post-stimulus BOLD undershoot if there is a post-stimulus undershoot in CBF and total CBV.

The third experiment offered positive and negative neuronal and BOLD responses to 20 s long visual stimuli overlapping and non-overlapping with the receptive field of the voxels in the ROI (Shmuel et al., 2006). The electrophysiological recordings of neuronal activity in V1 demonstrated that the positive neuronal responses can indeed exhibit a very pronounced response adaptation (but see below) and significant deactivation/activation during SP and PSP, respectively, similarly as estimated by P-DCM from the hemodynamic responses in the two experiments above. By modeling the dynamic changes in E-I balance during both SP and PSP, P-DCM was also able to explain the negative neuronal response, including the post-stimulus increase in neuronal activity. Note that this was achieved under the assumption that the input arriving to the excitatory population in V1 from LGN is negative (i.e., already LGN exhibits negative response, see e.g., Gouws et al., 2014). Furthermore, the relationship between the positive neuronal and BOLD responses in this experimental data is very interesting as there is a strong adaptation in the neuronal response during SP but no sign of adaptation in the BOLD response. This seems to appear as a typical observation for the BOLD responses to stimuli with a comparable stimulus duration measured in the V1 area of anesthetized macaque monkey brain (Logothetis et al., 2001; Logothetis, 2002; Pfeuffer et al., 2004; Goense and Logothetis, 2006) but less common in anesthetized cats or rats (as shown above and, e.g., Zhao et al., 2007; Kida and Yamamoto, 2008; Kim et al., 2010). Moreover, the stronger neuronal deactivation during PSP invites the hypothesis that the post-stimulus BOLD response undershoot could be related to this decrease in neuronal activity. However, the estimate of CBF response provided by P-DCM suggests that the neuronal adaptation and post-stimulus deactivations are almost entirely smoothed out by a slow rate of NVC (with χ = 0.19 Hz), which is also necessary to explain the smoothness of the observed BOLD response. This is in line with experimental observations from anesthetized macaque monkey brain reported in Pfeuffer et al. (2004), Zappe et al. (2008), and Zaldivar et al. (2014), albeit not explicitly described by the authors (see also discussion below for possible effects of anesthesia). Thus, in contrary to suggestion made in Shmuel et al. (2006), our modeling results suggest that the post-stimulus BOLD undershoot is not caused by the neuronal deactivation but by the vascular uncoupling (with τ_*PSP*_ ≅ 29 s) with a slow return of venous CBV to baseline during PSP (but see also below). Note that this vascular uncoupling is smaller than in the first two experiments, which is in a good agreement with the fact that the size of the post-stimulus undershoot and thus also size of the vascular uncoupling is proportional to stimulus duration (Uludağ and Blinder, 2017).

On the other hand, the shape of the negative BOLD response follows very closely the shape of the negative neuronal response with a smaller delay of the negative peak with respect to the stimulus onset and the post-stimulus overshoot to stimulus offset compared to the positive BOLD response. The faster evolution of the negative BOLD response (with an earlier post-stimulus undershoot) compared to the positive BOLD response was also observed in other studies using human subjects (Shmuel et al., 2002; Pfeuffer et al., 2004; Huber et al., 2014a). As we assumed that the passive hemodynamic properties of the venous compartment are the same for both positive and negative responses (including the size of vascular uncoupling), P-DCM explained the dynamic relationship between neuronal and BOLD response by a faster NVC (with χ = 0.85 Hz) for decreases in neuronal activity. In contrast to the positive BOLD response, the neuronal transients are reflected in the BOLD signal time-course due to the fact that the relative change of neuronal amplitude between stimulus onset and offset is larger (close to baseline by the end of the stimulation) compared to the positive response. Note that theoretically, one could also explain the discrepancy between positive neuronal and BOLD responses by making the NVC faster, increasing the mean transit time (*t*_0_) and minimizing the vascular uncoupling (τ), which would result in the post-stimulus BOLD undershoot having mainly neuronal origin. However, this would lead to *t*_0_ > 4.5 *s*, which would have to significantly differ from the negative BOLD response. That is, we would not be able to assume the same hemodynamic (i.e., vascular) model for both negative and positive BOLD response in the same voxels, which is not physiologically plausible. In general, it is more likely that the main differences between the positive and negative BOLD responses are due to different control of NVC (Lauritzen, 2005). Whether the rate difference in NVC for positive and negative responses is a general distinctive feature will need to be clarified in future experiments.

In summary, the three experimental data-sets provided physiological measurements at different stages of the dynamic cascade between neuronal and BOLD responses. First, we demonstrated that P-DCM was able to estimate (excitatory and inhibitory) neuronal responses with different amount of response adaptation during the stimulus period and post-stimulus deactivation/activation after the stimulation. We showed that the response adaptation during SP can vary from fast and strong (e.g., in the third experiment) to minimal (e.g., in the first experiment, response to flickering stimulus). Similarly, during PSP, the excitatory neuronal activity can slowly return to baseline (e.g., in the first experiment, after the response to the static stimulus) or it can decrease below baseline following the positive response (e.g., in the second experiment) or increase above baseline following the negative response. Second, the CBF response was shown to reflect the neuronal time-courses in a smoothed fashion via feedforward NVC, which can reduce or even completely eliminate neuronal transients (as in the third experiment for the positive CBF response). However, modeling of the NVC allows recovering excitatory and inhibitory neuronal transients from the CBF data. Additional transient phenomena in the BOLD response are induced by CBF-venous CBV uncoupling. That is, the discrepancy between the BOLD signal and CBF are due to venous CBV (or, alternatively, CMRO_2_, but see Havlicek et al., 2017a). That is, the presence or absence of dynamic features in the BOLD signal is not an unambiguous indication of the presence or absence of those features on the neuronal level. However, P-DCM applied to multi-modal data was able to dissociate between neuronal and vascular contributions to the BOLD response transients induced by different types and durations of stimuli. Furthermore, P-DCM accommodated the magnetic field strength and sequence parameters differences between experimental studies that also influence the size and nonlinearity of BOLD response transients (Havlicek et al., 2015, 2017a; Uludağ and Blinder, 2017). We think that P-DCM and its emphasis on response transients may be useful to also explain other combinations of multimodal data (e.g., neuronal activity recordings, CBF, CBV and BOLD) or to play an important role in combining EEG and fMRI data (Valdes Sosa et al., 2009; Riera and Sumiyoshi, 2010; Rosa et al., 2011; Butler et al., 2017; Friston et al., 2017). The neuronal mass activity of post-synaptic signals can be decomposed to non-overlapping frequency bands. The higher frequency band (gamma) is more associated with the main signal change of the hemodynamic response while lower frequency bands, such as alpha, and beta carry more information about changes in response transients (Magri et al., 2012; Mullinger et al., 2013, 2014; Ding et al., 2016).

Finally, it is important to realize that due to complexity of physiological mechanisms transforming the neuronal response to the BOLD response, the standard linear analysis (e.g., Friston et al., 1995, 1998) or (linear) deconvolution applied to BOLD data (e.g., Gaudes et al., 2011; Ryali et al., 2011; Smith et al., 2012; Bush and Cisler, 2013) cannot provide a reliable estimate of the underlying changes in neuronal activity in any of the three experiments. Our results highlight the necessity of nonlinear models, such as P-DCM, which account for dynamic uncoupling at both the neuronal and vascular levels and that can benefit from multi-modal data. In addition, nonlinear generative models, such as P-DCM, have the potential also to improve novel data analysis approaches, e.g., single-voxel and multi-voxel-pattern or representational similarity analysis approaches (e.g., Kriegeskorte et al., 2008; Haxby, 2012).

### Model assumptions and limitations

It is generally believed that modeling E-I balance—as the underlying source of the BOLD response (Logothetis, 2008)—is crucial for relating neuronal and hemodynamic responses. The applied neuronal model of E-I balance within P-DCM represents a large simplification of the underlying complex neuronal processes that operate at very fine temporal and spatial scales. The aim of this model is to mainly characterize regional post-synaptic changes in excitatory and inhibitory activity using a simple mathematical function that can be related to changes in the hemodynamic signal. It is assumed that the two-state neuronal model is driven by an exogenous input entering the excitatory state and the change in excitatory activity is followed by a (smaller) change in the inhibitory activity that subsequently modulates the excitatory activity via negative feedback and the E-I (im)balance eventually settles into a new balance state. Despite its simplicity, this model is able to represent dynamic change in E-I balance resulting in wide repertoire of neuronal response adaptions profiles (Hoge et al., 1999; Bandettini and Ungerleider, 2001; Logothetis et al., 2001), including more abrupt changes of E-I balance with stimulus cessation followed persistence of inhibitory activity that creates the post-stimulus deactivation (Sadaghiani et al., 2009; Mullinger et al., 2013, 2014, 2017). Therefore, P-DCM offers a new way to assess the neuronal origin of hemodynamic response transients by means of proxies for excitatory and inhibitory responses that can have high neuroscientific relevance.

Changes in the E-I balance were encoded in three neuronal parameters (σ, μ, λ). The temporal evolution of excitatory and inhibitory activity, including their dynamic mismatch, was controlled by the parameters σ and λ, respectively, and the strength of the inhibitory activity modulating the excitatory activity is encoded by the parameter μ. In this paper, we have mostly commented on the shape of estimated neuronal response and emphasized possible differences in excitatory and inhibitory dynamics. However, the actual values of estimated parameters controlling the neuronal model are informative and significantly differed between the three experiments (especially σ and μ). It is possible that these differences can be attributed to the neuronal or stimulus properties, as described above (Kida and Yamamoto, 2008), but also to the fact that responses of the two animal experiments were acquired under anesthesia (see below). In addition, testing for significant differences in estimated neuronal parameters due to experimental modulation or between healthy and diseased subjects is potentially an important area for future P-DCM utilization (Stephan et al., 2017). Note that in this paper we have made the specific assumption that (σ, μ) can vary between both SP and PSP but also between experimental conditions. On the other hand, λ was allowed to vary only between experimental conditions. This is because we favored a simpler model that was able to explain the observed neuronal responses in all three experiments, even with λ being only condition specific. Additionally, in the first experiment, the CBF response during PSP exhibits a slower return to the baseline without post-stimulus undershoot. This is effectively modeled by setting the parameter μ (i.e., the influence of inhibitory to excitatory activity) close to zero, which means that during this PSP, parameter λ does not have an effect on the shape of the neuronal response and becomes unidentifiable. Nevertheless, the inhibitory responses (as displayed in Figures 2–4) are modulated by the parameter μ, which makes them time-period specific as well.

Furthermore, the assumption about the input entering the excitatory state is appropriate for the majority of cortical regions as the vast majority of long-range connections between regions are mediated by excitatory neurons (Markram et al., 2004). For an additional description how P-DCM can model long-range connections, please see Supplementary Material 3 in Havlicek et al. (2015). We have utilized the same assumption also for the negative neuronal response but with a negative input entering the excitatory state, as it was shown earlier that negative responses in primary visual areas may be preceded by negative responses in LGN (e.g., Gouws et al., 2014).

NVC in P-DCM transforms neuronal to CBF response using a feedforward mechanism. While the motivation for utilizing feedforward NVC is fully discussed in Havlicek et al. (2015), we have also demonstrated in Havlicek et al. (2017b) that NVC based on negative feedback mechanism (Friston et al., 2000) is suboptimal and that the feedforward NVC in conjunction with the two-state neuronal model of E-I balance is preferred for modeling fMRI data. NVC is controlled by three parameters (φ, ϕ, χ), but optimizing only χ is sufficient to adjust the smoothness and delay of CBF response with respect to the neuronal response (i.e., we prefer a parsimonious NVC model with a minimum number of free parameters). The NVC parameter χ was assumed to vary between conditions. This is mainly because the third experiment involved a condition resulting in the negative response, and it was suggested earlier that the NVC of positive and negative hemodynamic response may differ (see e.g., Lauritzen, 2005; Huber et al., 2014a), which is supported here by our results obtained with constrained model inversion by multi-modal data. In fact, our fitting results of the first experiment showed that positive hemodynamic responses, although resulting from two different stimulus types and having different modulation of response transients, can have the same NVC parameter χ (see Table 2).

In the hemodynamic model, P-DCM assumes that CBF and CMRO_2_ are tightly coupled. There are three reasons to do so: (1) It is a common assumption in DCM literature that CBF is tightly coupled with CMRO_2_ (Friston et al., 2003), and other papers showed that CBF and venous CBV are uncoupled (e.g., Mandeville et al., 1999; Kim and Kim, 2011; Huber et al., 2014a), as for longer stimuli venous CBV response exhibits much slower increase and return to baseline compared to the CBF response; (2) If one considers both CMRO_2_ and venous CBV responses uncoupled from CBF response, then both can have similar impact on the transients of the BOLD response and the generative model becomes unidentifiable. From modeling (and model inversion) perspective, this is seen as redundancy and therefore it is preferable to fix one of these two mechanisms. This is because under normal conditions even with multimodal data (consisting of CBF, total CBV and BOLD) one cannot effectively disentangle these two mechanisms from each other; (3) We have recently provided a comprehensive proof using multi-echo data (Havlicek et al., 2017a) that next to CBF (i.e., neuronal) contribution, the CBF-venous CBV uncoupling (and not the CBF-CMRO_2_ uncoupling) is the mechanism behind the BOLD response transients. In this case, the specific echo-time dependence of the BOLD response transients (related to contribution from both extravascular and intravascular signals) together with a variable CBF response allowed us to identify the underlying mechanism.

In this paper, we have selected three multi-modal data-sets as illustrative examples to demonstrate the versatility of P-DCM to explain underlying causal relationships under the constraint of multiple physiological measurements. We aimed to include multi-modal data-sets acquired at different magnetic field strengths, containing different combinations of physiological variables next to the standard BOLD response, and possibly involving more than one level of experimental manipulation. Further, we favored averaged data with excellent signal-to-noise quality, which clearly manifest discrepancy between different physiological variables and between experimental conditions. There are certainly many more interesting published multi-modal data-sets that P-DCM could be further tested on. For example, we have limited our demonstration to human, monkey and cat data, however, there are many excellent multi-modal data measured in rodents (e.g., Kida et al., 2007; Boorman et al., 2010; Füchtemeier et al., 2010; Hyder et al., 2010; Hirano et al., 2011). Since a good correspondence was shown earlier between hemodynamic responses measured in cats and rats (see e.g., Zong et al., 2012) and in monkeys and rats (see e.g., Huber et al., 2015), we expect that P-DCM could perform well also if applied to rodent data. Furthermore, as model parameters are specified in terms of priors, it is also possible to account for interspecies differences in physiological parameters. For example, a higher baseline blood flow and volume influences the main transit time, which could be adjusted for different species (even though it was not necessary in our case, as the mean transit time was one of the free parameters).

All three data-sets represent evoked responses to longer sustained stimuli measured in the primary visual cortex. This choice allowed us to consider model assumptions that could be shared between all three experiments (as mentioned above) and fitting results obtained from these experiments could be more directly compared with each other. Additionally, in our previous aforementioned study (Havlicek et al., 2017a), the same static and flickering stimuli, including identical stimulus durations, provided high evidence that the BOLD response transients are mainly of neuronal and vascular origin (with negligible or zero contribution of CBF-CMRO_2_ uncoupling). Therefore, we are in a good position to extend these results to the model assumptions applied in this paper, especially in the case of the first experiment. Similarly, the authors of the second experiment data-set showed in the independent study that with the same type of stimulus, the venous CBV response in the primary visual cortex of the cat exhibits much slower dynamics compared to the arterial CBV (Kim and Kim, 2011). Furthermore, other multi-modal data acquired in the primary visual cortex of monkey brain (i.e., comparable to the third experiment) showed that despite deactivation of after stimulus cessation in the neuronal response, no or negligible post-stimulus undershoot was present in the CBF response but significantly present in the BOLD response (Pfeuffer et al., 2004; Zappe et al., 2008; Zaldivar et al., 2014). This supports our assumption and fitting result that CBF-venous CBV uncoupling can play important role in explaining the observed BOLD response transients also in third experiment. In general, the selected data may have revealed limitations of the structure of P-DCM and assumptions of specific parameters in the generative model. However, as the generative model was able to reproduce the experimental observations, P-DCM proves to be flexible enough to accommodate a wide range of multi-modal experimental data. Nevertheless, more work on novel data (including short stimuli and other brain regions) must be performed to further evaluate and develop current generative model used in P-DCM.

Finally, the second and third experiments were performed on anesthetized animals using isoflurane (i.e., a common anesthetic agent used in animal research). In general, anesthesia is known to influence the amplitude and shape of both neuronal and hemodynamic responses (Krautwald and Angenstein, 2012; Uludağ and Blinder, 2017). Under anesthesia using isoflurane, the neuronal baseline (i.e., the firing rate) is decreased and the neuronal responses exhibit smaller changes and can have more pronounced and faster adaptation compared to the awake state (Aksenov et al., 2015; Keller et al., 2017). This can potentially explain the large adaptation profiles of the neuronal responses in the second and third experiment, even though dynamic stimuli were applied (i.e., one would expect responses more comparable to responses to flickering rather than static stimuli in the first experiment). Having said this, larger differences in the shape of neuronal responses due to experimental modulations are expected during awake state (Haider et al., 2013; Bahmani et al., 2014; Keller et al., 2017), highlighting some benefits of human fMRI over animal studies under anesthesia. At the hemodynamic or NVC level, isoflurane based anesthesia leads to vasodilatation of mainly arteries and arterioles in the occipital areas, which results in increase of baseline CBF but even larger increase of baseline CBV; i.e., the mean transit time (in the microvasculature) is increased as well (Lorenz et al., 2001). Subsequently, the change in CBF due to activation is smaller and more delayed compared to awake state (Sicard et al., 2003; Pisauro et al., 2013). As a result, BOLD responses during anesthesia are also smaller. The anesthesia is expected to influence more the active mechanisms of the arterial compartment (in our model represented by CBF) than the passive mechanism of the venous compartment. Note that, theoretically, the same type of anesthetic agents could also have different effect on the neuronal responses and NVC between species, which could explain the large discrepancy between the second and third experiment (besides the obvious differences in stimulus type and stimulus duration). As P-DCM is able to optimize all stages of the physiological chain from the neuronal to the hemodynamic responses, it can also be useful in characterizing the differences in the physiological mechanisms during both anesthesia and the awake state.

## Author contributions

MH, KU, AR, and DI designed the experiment. MH and DI collected the data. MH and KU designed and performed data analysis. MH performed model inversions and created figures. MH, DI, AR, and KU wrote and revised the manuscript. All authors approved the final manuscript.

### Conflict of interest statement

The authors declare that the research was conducted in the absence of any commercial or financial relationships that could be construed as a potential conflict of interest.

## Footnotes

^1^As we have developed our novel generative model within the framework of the dynamic causal model (DCM) and applied it both to single ROI and to a network of ROIs, in this paper to avoid confusion, we use DCM and the generative model embedded within P-DCM interchangeably.

^2^Please note that we use PSP instead of PSU, as the sign of the post-stimulus BOLD response depends on whether it is preceded by negative or positive changes in neuronal activity during stimulation (as in Shmuel et al., 2006; whose data are investigated in the current study).

^3^This is the reason why we extended the stimulation period for negative response by 1 s.

## References

[B1] Aksenov D. P. Li L. Miller M. J. Iordanescu G. Wyrwicz A. M. (2015). Effects of anesthesia on BOLD signal and neuronal activity in the somatosensory cortex. J. Cereb. Blood Flow Metab. 35, 1819–1826. 10.1038/jcbfm.2015.130 26104288 PMC4635237

[B2] Attwell D. Buchan A. M. Charpak S. Lauritzen M. MacVicar B. A. Newman E. A. (2010). Glial and neuronal control of brain blood flow. Nature 468, 232–243. 10.1038/nature09613 21068832 PMC3206737

[B3] Attwell D. Iadecola C. (2002). The neural basis of functional brain imaging signals. Trends Neurosci. 25, 621–625. 10.1016/S0166-2236(02)02264-6 12446129

[B4] Bahmani H. Murayama Y. Logothetis N. K. Keliris G. A. (2014). Binocular flash suppression in the primary visual cortex of anesthetized and awake macaques. PloS ONE 9:e107628. 10.1371/journal.pone.0107628 25216188 PMC4162631

[B5] Bandettini P. A. Ungerleider L. G. (2001). From neuron to BOLD: new connections. Nat. Neurosci. 4, 49–51. 10.1038/nn0901-864 11528412

[B6] Birn R. M. Saad Z. S. Bandettini P. A. (2001). Spatial heterogeneity of the nonlinear dynamics in the FMRI BOLD response. Neuroimage 14, 817–826. 10.1006/nimg.2001.0873 11554800

[B7] Boorman L. Kennerley A. J. Johnston D. Jones M. Zheng Y. Redgrave P.. (2010). Negative blood oxygen level dependence in the rat: a model for investigating the role of suppression in neurovascular coupling. J. Neurosci. 30, 4285–4294. 10.1523/JNEUROSCI.6063-09.2010 20335464 PMC6634501

[B8] Boynton G. M. (2011). Spikes, BOLD, attention, and awareness: a comparison of electrophysiological and fMRI signals in V1. J. Vis. 11:12 10.1167/11.5.12 22199162 PMC4124818

[B9] Boynton G. M. Engel S. A. Glover G. H. Heeger D. J. (1996). Linear systems analysis of functional magnetic resonance imaging in human V1. J. Neurosci. 16, 4207–4221. 8753882 10.1523/JNEUROSCI.16-13-04207.1996 PMC6579007

[B10] Bush K. Cisler J. (2013). Decoding neural events from fMRI BOLD signal: a comparison of existing approaches and development of a new algorithm. Magn. Reson. Imaging 31, 976–989. 10.1016/j.mri.2013.03.015 23602664 PMC3738068

[B11] Butler R. Bernier P.-M. Lefebvre J. Gilbert G. Whittingstall K. (2017). Decorrelated input dissociates narrow band gamma power and BOLD in human visual cortex. J. Neurosci. 37, 5408–5418. 10.1523/JNEUROSCI.3938-16.2017 28455370 PMC6596528

[B12] Buxton R. B. Miller K. Frank L. R. Wong E. C. (1998a). BOLD signal dynamics: the balloon model with viscoelastic effects, in Proceeding of ISMRM Conference (Sydney, NSW), 1.

[B13] Buxton R. B. Wong E. C. Frank L. R. (1998b). Dynamics of blood flow and oxygenation changes during brain activation: the balloon model. Magn. Res. Med. 39, 855–864. 10.1002/mrm.1910390602 9621908

[B14] Cauli B. Hamel E. (2010). Revisiting the role of neurons in neurovascular coupling. Front. Neuroenergetics 2:9. 10.3389/fnene.2010.00009 20616884 PMC2899521

[B15] Chen J. J. Pike G. B. (2009). Origins of the BOLD post-stimulus undershoot. Neuroimage 46, 559–568. 10.1016/j.neuroimage.2009.03.015 19303450

[B16] Devor A. Bandettini P. A. Boas D. A. Bower J. M. Buxton R. B. Cohen L. B.. (2013). The challenge of connecting the dots in the B.R.A.I.N. Neuron 80, 270–274. 10.1016/j.neuron.2013.09.008 24139032 PMC3864648

[B17] Devor A. Tian P. Nishimura N. Teng I. C. Hillman E. M. C. Narayanan S. N.. (2007). Suppressed neuronal activity and concurrent arteriolar vasoconstriction may explain negative blood oxygenation level-dependent signal. J. Neurosci. 27, 4452–4459. 10.1523/JNEUROSCI.0134-07.2007 17442830 PMC2680207

[B18] Ding N. Simon J. Z. Shamma S. A. David S. V. (2016). Encoding of natural sounds by variance of the cortical local field potential. J. Neurosci. 115, 2389–2398. 10.1152/jn.00652.2015 26912594 PMC4922460

[B19] Donahue M. J. Lu H. Jones C. K. Pekar J. J. Zijl P. C. M. V. (2006). An account of the discrepancy between MRI and PET cerebral blood flow measures. A high-field MRI investigation. NMR Biomed. 19, 1043–1054. 10.1002/nbm.1075 16948114

[B20] Donahue M. J. Stevens R. D. de Boorder M. Pekar J. J. Hendrikse J. van Zijl P. C. M. (2009). Hemodynamic changes after visual stimulation and breath holding provide evidence for an uncoupling of cerebral blood flow and volume from oxygen metabolism. J. Cereb. Blood Flow Metab. 29, 176–185. 10.1038/jcbfm.2008.109 18797471 PMC2865199

[B21] Drew P. J. Shih A. Y. Kleinfeld D. (2011). Fluctuating and sensory-induced vasodynamics in rodent cortex extend arteriole capacity. Proc. Natl. Acad. Sci. U.S.A. 108, 8473–8478. 10.1073/pnas.1100428108 21536897 PMC3100929

[B22] Frahm J. Baudewig J. Kallenberg K. Kastrup A. Merboldt K. D. Dechent P. (2008). The post-stimulation undershoot in BOLD fMRI of human brain is not caused by elevated cerebral blood volume. Neuroimage 40, 473–481. 10.1016/j.neuroimage.2007.12.005 18201912

[B23] Frahm J. Krüger G. Merboldt K. D. Kleinschmidt A. (1996). Dynamic uncoupling and recoupling of perfusion and oxidative metabolism during focal brain activation in man. Magn. Reson. Med. 35, 143–148. 10.1002/mrm.1910350202 8622575

[B24] Friston K. J. Fletcher P. Josephs O. Holmes A. Rugg M. D. Turner R. (1998). Event-related fMRI: characterizing differential responses. Neuroimage 7, 30–40. 10.1006/nimg.1997.0306 9500830

[B25] Friston K. J. Harrison L. Penny W. (2003). Dynamic causal modelling. Neuroimage 19, 1273–1302. 10.1016/S1053-8119(03)00202-7 12948688

[B26] Friston K. J. Mechelli A. Turner R. Price C. J. (2000). Nonlinear responses in fMRI: the balloon model, volterra kernels, and other hemodynamics. NeuroImage 12, 466–477. 10.1006/nimg.2000.0630 10988040

[B27] Friston K. J. Preller K. H. Mathys C. Cagnan H. Heinzle J. Razi A.. (2017). Dynamic causal modelling revisited. NeuroImage. [Epub ahead of print]. 10.1016/j.neuroimage.2017.02.045 28219774 PMC6693530

[B28] Friston K. J. Holmes A. P. Worsley K. J. Poline J. P. Frith C. D. Frackowiak R. S. J. (1995). Statistical parametric maps in functional imaging: a general linear approach. Hum. Brain Mapp. 2, 189–210. 10.1002/hbm.460020402

[B29] Friston K. Mattout J. Trujillo-Barreto N. Ashburner J. Penny W. (2007). Variational free energy and the Laplace approximation. Neuroimage 34, 220–234. 10.1016/j.neuroimage.2006.08.035 17055746

[B30] Füchtemeier M. Leithner C. Offenhauser N. Foddis M. Kohl-Bareis M. Dirnagl U.. (2010). Elevating intracranial pressure reverses the decrease in deoxygenated hemoglobin and abolishes the post-stimulus overshoot upon somatosensory activation in rats. Neuroimage 52, 445–454. 10.1016/j.neuroimage.2010.04.237 20420930

[B31] Gagnon L. SakadŽić S. Lesage F. Musacchia J. J. Lefebvre J. Fang Q.. (2015). Quantifying the microvascular origin of BOLD-fMRI from first principles with two-photon microscopy and an oxygen-sensitive nanoprobe. J. Neurosci. 35, 3663–3675. 10.1523/JNEUROSCI.3555-14.2015 25716864 PMC4339366

[B32] Gaudes C. Petridou N. Dryden I. L. Bai L. Francis S. T. Gowland P. A. (2011). Detection and characterization of single-trial fMRI BOLD responses: paradigm free mapping. Hum. Brain Mapp. 1418, 1400–1418. 10.1002/hbm.21116 PMC6869937 20963818

[B33] Goense J. B. Logothetis N. K. (2006). Laminar specificity in monkey V1 using high-resolution SE-fMRI. Magn. Reson. Imaging 24, 381–392. 10.1016/j.mri.2005.12.032 16677944

[B34] Goense J. B. M. Logothetis N. K. (2008). Neurophysiology of the BOLD fMRI signal in awake monkeys. Curr. Biol. 18, 631–640. 10.1016/j.cub.2008.03.054 18439825

[B35] Gouws A. D. Alvarez I. Watson D. M. Uesaki M. Rodgers J. Morland A. B. (2014). On the role of suppression in spatial attention: evidence from negative BOLD in human subcortical and cortical. J. Neurosci. 34, 10347–10360. 10.1523/JNEUROSCI.0164-14.2014 25080595 PMC6608280

[B36] Grill-Spector K. Malach R. (2001). fMR-adaptation: a tool for studying the functional properties of human cortical neurons. Acta Psychol. (Amst) 107, 293–321. 10.1016/S0001-6918(01)00019-1 11388140

[B37] Grubb R. L. Raichle M. E. Eichling J. O. Ter-Pogossian M. M. (1974). The effects of changes in PaCO_2_ cerebral blood volume, blood flow, and vascular mean transit time. Stroke 5, 630–639. 10.1161/01.STR.5.5.630 4472361

[B38] Haider B. Häusser M. Carandini M. (2013). Inhibition dominates sensory responses in the awake cortex. Nature 493, 2–8. 10.1038/nature12370 23172139 PMC3537822

[B39] Havlicek M. Ivanov D. Poser B. A. Uludağ K. (2017a). Echo-time dependence of the BOLD response transients–a window into brain functional physiology. Neuroimage 159, 355–370. 10.1016/j.neuroimage.2017.07.034 28729160

[B40] Havlicek M. Roebroeck A. Friston K. Gardumi A. Ivanov D. Uludağ K. (2015). Physiologically informed dynamic causal modeling of fMRI data. Neuroimage 122, 355–372. 10.1016/j.neuroimage.2015.07.078 26254113

[B41] Havlicek M. Roebroeck A. Friston K. J. Gardumi A. Ivanov D. Uludağ K. (2017b). On the importance of modeling fMRI transients when estimating e ff ective connectivity: A dynamic causal modeling study using ASL data. NeuroImage 155, 217–233. 10.1016/j.neuroimage.2017.03.017 28323165

[B42] Haxby J. V. (2012). NeuroImage multivariate pattern analysis of fMRI: The early beginnings. Neuroimage 62, 852–855. 10.1016/j.neuroimage.2012.03.016 22425670 PMC3389290

[B43] Hillman E. M. (2014). Coupling mechanism and significance of the BOLD signal: a status report. Annu. Rev. Neurosci. 37, 161–181. 10.1146/annurev-neuro-071013-014111 25032494 PMC4147398

[B44] Hirano Y. Stefanovic B. Silva A. C. (2011). Spatiotemporal evolution of the functional magnetic resonance imaging response to ultrashort stimuli. J. Neurosci. 31, 1440–1447. 10.1523/JNEUROSCI.3986-10.2011 21273428 PMC3078723

[B45] Hoge R. D. Atkinson J. Gill B. Crelier G. R. Marrett S. Pike G. B. (1999). Stimulus-dependent BOLD and perfusion dynamics in human V1. NeuroImage 9(6 Pt 1), 573–585. 10.1006/nimg.1999.0443 10334901

[B46] Hua J. Stevens R. D. Huang A. J. Pekar J. J. Zijl P. C. M. V. (2011). Physiological origin for the BOLD poststimulus undershoot in human brain: vascular compliance versus oxygen metabolism. J. Cereb. Blood Flow Metab. 31, 1599–1611. 10.1038/jcbfm.2011.35 21468090 PMC3137471

[B47] Huber L. Goense J. Kennerley A. J. Ivanov D. Krieger S. N. Lepsien J. (2014a). Investigation of the neurovascular coupling in positive and negative BOLD responses in human brain at 7T. Neuroimage 97, 349–362. 10.1016/j.neuroimage.2014.04.022 24742920

[B48] Huber L. Goense J. Kennerley A. J. Trampel R. Guidi M. Reimer E. (2015). Cortical lamina-dependent blood volume changes in human brain at 7T. Neuroimage 107, 23–33. 10.1016/j.neuroimage.2014.11.046 25479018

[B49] Huber L. Ivanov D. Krieger S. N. Streicher M. N. Mildner T. Poser B. A.. (2014b). Slab-selective, BOLD-corrected VASO at 7 Tesla provides measures of cerebral blood volume reactivity with high signal-to-noise ratio. Magn. Reson. Med. 72, 137–148. 10.1002/mrm.24916 23963641

[B50] Hyder F. Sanganahalli B. G. Herman P. Coman D. Maandag N. J. G. Behar K. L.. (2010). Neurovascular and neurometabolic couplings in dynamic calibrated fMRI: transient oxidative neuroenergetics for block-design and event-related paradigms. Front. Neuroenergetics 2:18. 10.3389/fnene.2010.00018 20838476 PMC2936934

[B51] Jin T. Kim S.-G. (2008). Cortical layer-dependent dynamic blood oxygenation, cerebral blood flow and cerebral blood volume responses during visual stimulation. Neuroimage 43, 1–9. 10.1016/j.neuroimage.2008.06.029 18655837 PMC2579763

[B52] Keller A. J. Houlton R. Kampa B. M. Lesica N. A. Mrsic-flogel T. D. Keller G. B.. (2017). Stimulus relevance modulates contrast adaptation in visual cortex. Elife 6, 4–15. 10.7554/eLife.21589 28130922 PMC5298877

[B53] Kida I. Rothman D. L. Hyder F. (2007). Dynamics of changes in blood flow, volume, and oxygenation: implications for dynamic functional magnetic resonance imaging calibration. J. Cereb. Blood Flow Metab. 27, 690–696. 10.1038/sj.jcbfm.9600409 17033688 PMC2854582

[B54] Kida I. Yamamoto T. (2008). Stimulus frequency dependence of blood oxygenation level-dependent functional magnetic resonance imaging signals in the somatosensory cortex of rats. Neurosci. Res. 62, 25–31. 10.1016/j.neures.2008.05.006 18602178

[B55] Kim D.-S. Ronen I. Olman C. Kim S.-G. Ugurbil K. Toth L. J. (2004). Spatial relationship between neuronal activity and BOLD functional MRI. Neuroimage 21, 876–885. 10.1016/j.neuroimage.2003.10.018 15006654

[B56] Kim S.-G. (1995). Quantification of relative cerebral blood flow change by flow-sensitive alternating inversion recovery (FAIR) technique: application to functional mapping. Magn. Reson. Med. 34, 293–301. 10.1002/mrm.1910340303 7500865

[B57] Kim S.-G. Ogawa S. (2012). Biophysical and physiological origins of blood oxygenation level-dependent fMRI signals. J. Cereb. Blood Flow Metab. 32, 1188–1206. 10.1038/jcbfm.2012.23 22395207 PMC3390806

[B58] Kim T. Kim S.-G. (2011). Temporal dynamics and spatial specificity of arterial and venous blood volume changes during visual stimulation: implication for BOLD quantification. J. Cereb. Blood Flow Metab. 31, 1211–1222. 10.1038/jcbfm.2010.226 21179068 PMC3099637

[B59] Kim T. Masamoto K. Fukuda M. Vazquez A. Kim S.-G. (2010). Frequency-dependent neural activity, CBF, and BOLD fMRI to somatosensory stimuli in isoflurane-anesthetized rats. Neuroimage 52, 224–233. 10.1016/j.neuroimage.2010.03.064 20350603 PMC2883664

[B60] Krautwald K. Angenstein F. (2012). Low frequency stimulation of the perforant pathway generates anesthesia-specific variations in neural activity and BOLD responses in the rat dentate gyrus. J. Cereb. Blood Flow Metab. 32, 291–305. 10.1038/jcbfm.2011.126 21863039 PMC3272596

[B61] Krekelberg B. Boynton G. M. van Wezel R. J. A. (2006). Adaptation: from single cells to BOLD signals. Trends Neurosci. 29, 250–256. 10.1016/j.tins.2006.02.008 16529826

[B62] Kriegeskorte N. Mur M. Bandettini P. (2008). Representational similarity analysis–connecting the branches of systems neuroscience. Front. Syst. Neurosci. 2:4. 10.3389/neuro.06.004.2008 19104670 PMC2605405

[B63] Krüger G. Kleinschmidt A. Frahm J. (1996). Dynamic MRI sensitized to cerebral blood oxygenation and flow during sustained activation. Magn. Reson. Med. 35, 4. 10.1002/mrm.1910350602 8744004

[B64] Lauritzen M. (2005). Reading vascular changes in brain imaging: is dendritic calcium the key? Nature reviews. Neuroscience 6, 77–85. 10.1038/nrn1589 15611729

[B65] Liu T. T. Brown G. G. (2007). Measurement of cerebral perfusion with arterial spin labeling: part 1. Methods. J. Int. Neuropsychol. Soc. 13, 517–525. 10.1017/S1355617707070646 17445301

[B66] Logothetis N. K. (2002). The neural basis of the blood–oxygen–level–dependent functional magnetic resonance imaging signal. Philos. Trans. R. Soc. Lond. Ser. B Biol. Sci. 357, 1003. 10.1098/rstb.2002.1114 12217171 PMC1693017

[B67] Logothetis N. K. (2008). What we can do and what we cannot do with fMRI. Nature 453, 869–878. 10.1038/nature06976 18548064

[B68] Logothetis N. K. Augath M. Murayama Y. Rauch A. Sultan F. Goense J.. (2010). The effects of electrical microstimulation on cortical signal propagation. Nat. Neurosci. 13, 1283–1291. 10.1038/nn.2631 20818384

[B69] Logothetis N. K. Pauls J. Augath M. Trinath T. Oeltermann A. (2001). Neurophysiological investigation of the basis of the fMRI signal. Nature 412, 150–157. 10.1038/35084005 11449264

[B70] Logothetis N. K. Wandell B. A. (2004). Interpreting the BOLD signal. Annu. Rev. Physiol. 66, 735–769. 10.1146/annurev.physiol.66.082602.092845 14977420

[B71] Lorenz I. H. Kolbitsch C. Hormann C. Luger T. J. Schocke M. Felber S. (2001). Inluence of equianaesthetic concentrations of nitrous oxide and isoflurane on regional cerebral blood flow, regional cerebral blood volume, and regional mean transit time in human volunteers. Br. J. Anaesth. 87, 691–698. 10.1093/bja/87.5.691 11878518

[B72] Lu H. Golay X. Pekar J. J. Van Zijl P. C. M. (2003). Functional magnetic resonance imaging based on changes in vascular space occupancy. Magn. Reson. Med. 50, 263–274. 10.1002/mrm.10519 12876702

[B73] Lu H. Golay X. Pekar J. J. Van Zijl P. C. M. (2004). Sustained poststimulus elevation in cerebral oxygen utilization after vascular recovery. J. Cereb. Blood Flow Metab. 24, 764–770. 10.1097/01.WCB.0000124322.60992.5C 15241184

[B74] Magri C. Schridde U. Murayama Y. Panzeri S. Logothetis N. K. (2012). The amplitude and timing of the BOLD signal reflects the relationship between local field potential power at different frequencies. J. Neurosc. 32, 1395–1407. 10.1523/JNEUROSCI.3985-11.2012 22279224 PMC6796252

[B75] Mandeville J. B. Marota J. J. A. Ayata C. Zaharchuk G. Moskowitz M. A. Rosen B. R.. (1999). Evidence of a cerebrovascular postarteriole windkessel with delayed compliance. J. Cereb. Blood Flow Metab. 19, 679–689. 10.1097/00004647-199906000-00012 10366199

[B76] Mandeville J. B. Marota J. J. Kosofsky B. E. Keltner J. R. Weissleder R. Rosen B. R.. (1998). Dynamic functional imaging of relative cerebral blood volume during rat forepaw stimulation. Magn. Reson. Med. 39, 615–624. 10.1002/mrm.1910390415 9543424

[B77] Markram H. Toledo-Rodriguez M. Wang Y. Gupta A. Silberberg G. Wu C. (2004). Interneurons of the neocortical inhibitory system. Nature reviews. Neuroscience 5, 793–807. 10.1038/nrn1519 15378039

[B78] Marreiros A. C. Kiebel S. J. Friston K. J. (2008). Dynamic causal modelling for fMRI: a two-state model. Neuroimage 39, 269–278. 10.1016/j.neuroimage.2007.08.019 17936017

[B79] Mayhew S. D. Mullinger K. J. Bagshaw A. P. Bowtell R. Francis S. T. (2014). Investigating intrinsic connectivity networks using simultaneous BOLD and CBF measurements. Neuroimage 99, 111–121. 10.1016/j.neuroimage.2014.05.042 24857826

[B80] Muckli L. (2010). What are we missing here? Brain imaging evidence for higher cognitive functions in primary visual cortex v1. Int. J. Imaging Syst. Technol. 20, 131–139. 10.1002/ima.20236

[B81] Mullinger K. J. Cherukara M. T. Buxton R. B. Francis S. T. Mayhew S. D. (2017). Post-stimulus fMRI and EEG responses: Evidence for a neuronal origin hypothesised to be inhibitory. Neuroimage 157, 388–399. 10.1016/j.neuroimage.2017.06.020 28610902 PMC6475192

[B82] Mullinger K. J. Mayhew S. D. Bagshaw A. P. Bowtell R. Francis S. T. (2013). Poststimulus undershoots in cerebral blood flow and BOLD fMRI responses are modulated by poststimulus neuronal activity. Proc. Natl. Acad. Sci. U.S.A. 110, 13636–13641. 10.1073/pnas.1221287110 23898206 PMC3746922

[B83] Mullinger K. J. Mayhew S. D. Bagshaw A. P. Bowtell R. Francis S. T. (2014). Evidence that the negative BOLD response is neuronal in origin: a simultaneous EEG-BOLD-CBF study in humans. Neuroimage 94, 263–274. 10.1016/j.neuroimage.2014.02.029 24632092

[B84] Mumford J. A. Hernandez-Garcia L. Lee G. R. Nichols T. E. (2006). Estimation efficiency and statistical power in arterial spin labeling fMRI. Neuroimage 33, 103–114. 10.1016/j.neuroimage.2006.05.040 16860577 PMC2772871

[B85] Niessing J. Ebisch B. Schmidt K. E. Niessing M. Singer W. Galuske R. A. W. (2005). Hemodynamic signals correlate tightly with synchronized gamma oscillations. Science 209, 948–951. 10.1126/science.1110948 16081740

[B86] Ogawa S. Lee T. M. Kay A. R. Tank D. W. (1990). Brain magnetic resonance imaging with contrast dependent on blood oxygenation. Proc. Natl. Acad. Sci. U.S.A. 87, 9868–9872. 10.1073/pnas.87.24.9868 2124706 PMC55275

[B87] Ozaki T. (1992). A bridge between nonlinear time series models and nonlinear stochastic dynamical systems: a local linearization approach. Stat. Sin. 2, 113–135.

[B88] Pérez-González D. Malmierca M. S. (2014). Adaptation in the auditory system: an overview. Front. Integr. Neurosci. 8:19. 10.3389/fnint.2014.00019 24600361 PMC3931124

[B89] Pfeuffer J. Merkle H. Beyerlein M. Steudel T. Logothetis N. K. (2004). Anatomical and functional MR imaging in the macaque monkey using a vertical large-bore 7 Tesla setup. Magn. Reson. Imaging 22, 1343–1359. 10.1016/j.mri.2004.10.004 15707785

[B90] Pisauro M. A. Dhruv N. T. Carandini M. Benucci A. (2013). Fast hemodynamic responses in the visual cortex of the awake mouse. J. Neurosci. 33, 18343–18351. 10.1523/JNEUROSCI.2130-13.2013 24227743 PMC3828474

[B91] Poser B. A. Mierlo E. V. Norris D. G. (2011). Exploring the post-stimulus undershoot with spin-echo fmri: implications for models of neurovascular response. Hum. Brain Mapp. 153, 141–153. 10.1002/hbm.21003 PMC6869868 20623748

[B92] Riera J. J. Sumiyoshi A. (2010). Brain oscillations: ideal scenery to understand the neurovascular coupling. Curr. Opin. Neurol. 23, 374–381. 10.1097/WCO.0b013e32833b769f 20610989

[B93] Rosa M. J. Kilner J. M. Penny W. D. (2011). Bayesian comparison of neurovascular coupling models using EEG-fMRI. PLoS Comput. Biol. 7:e1002070. 10.1371/journal.pcbi.1002070 21698175 PMC3116890

[B94] Ryali S. Supekar K. Chen T. Menon V. (2011). Multivariate dynamical systems models for estimating causal interactions in fMRI. Neuroimage 54, 807–823. 10.1016/j.neuroimage.2010.09.052 20884354 PMC2997172

[B95] Sadaghiani S. Ugurbil K. Uludağ K. (2009). Neural activity-induced modulation of BOLD poststimulus undershoot independent of the positive signal. Magn. Reson. Imaging 27, 1030–1038. 10.1016/j.mri.2009.04.003 19761930

[B96] Shmuel A. Augath M. Oeltermann A. Logothetis N. K. (2006). Negative functional MRI response correlates with decreases in neuronal activity in monkey visual area V1. Nat. Neurosci. 9, 569–577. 10.1038/nn1675 16547508

[B97] Shmuel A. Yacoub E. Pfeuffer J. Van de Moortele P. F. Adriany G. Hu X.. (2002). Sustained negative BOLD, blood flow and oxygen consumption response and its coupling to the positive response in the human brain. Neuron 36, 1195–1210. 10.1016/S0896-6273(02)01061-9 12495632

[B98] Sicard K. Shen Q. Brevard M. E. Sullivan R. Ferris C. F. King J. A.. (2003). Regional cerebral blood flow and BOLD responses in conscious and anesthetized rats under basal and hypercapnic conditions: implications for functional MRI studies. J. Cereb. Blood Flow Metab. 23, 472–481. 10.1097/01.WCB.0000054755.93668.20 12679724 PMC2989608

[B99] Smith J. F. Pillai A. Chen K. Horwitz B. Calhoun V. D. (2012). Effective connectivity modeling for fMRI: six issues and possible solutions using linear dynamic systems. Front. Syst. Neurosci. 5:104. 10.3389/fnsys.2011.00104 22279430 PMC3260563

[B100] Stephan K. E. Schlagenhauf F. Huys Q. J. M. Raman S. Aponte E. A. Brodersen K. H.. (2017). Computational neuroimaging strategies for single patient predictions. Neuroimage 145, 180–199. 10.1016/j.neuroimage.2016.06.038 27346545

[B101] Uludağ K. Dubowitz D. J. Yoder E. J. Restom K. Liu T. T. Buxton R. B. (2004). Coupling of cerebral blood flow and oxygen consumption during physiological activation and deactivation measured with fMRI. Neuroimage 23, 148–155. 10.1016/j.neuroimage.2004.05.013 15325361

[B102] Uludağ K. Müller-Bierl B. Ugurbil K. (2009). An integrative model for neuronal activity-induced signal changes for gradient and spin echo functional imaging. Neuroimage 48, 150–165. 10.1016/j.neuroimage.2009.05.051 19481163

[B103] Uludağ K. Blinder P. (2017). Linking brain vascular physiology to hemodynamic response in ultra-high field MRI. NeuroImage. [Epub ahead of print]. 10.1016/j.neuroimage.2017.02.063 28254456

[B104] Valdes Sosa P. A. Sanchez Bornot J. M. Sotero R. C. Iturria Medina Y. Aleman Gomez Y. Bosch Bayard J.. (2009). Model driven EEG/fMRI fusion of brain oscillations. Hum. Brain Mapp. 30, 2701–2721. 10.1002/hbm.20704 19107753 PMC6870602

[B105] van Zijl P. C. M. Hua J. Lu H. (2012). The BOLD post-stimulus undershoot, one of the most debated issues in fMRI. Neuroimage 62, 1092–1102. 10.1016/j.neuroimage.2012.01.029 22248572 PMC3356682

[B106] Wong E. C. Buxton R. B. Frank L. R. (1997). Implementation of quantitative perfusion imaging techniques for functional brain mapping using pulsed arterial spin labeling. NMR Biomed. 10, 237–249. 10.1002/(SICI)1099-1492(199706/08)10:4/5<237::AID-NBM475>3.0.CO;2-X 9430354

[B107] Yacoub E. Ugurbil K. Harel N. (2006). The spatial dependence of the poststimulus undershoot as revealed by high-resolution BOLD- and CBV-weighted fMRI. J. Cereb. Blood Flow Metab. 26, 634–644. 10.1038/sj.jcbfm.9600239 16222242

[B108] Zaldivar D. Rauch A. Whittingstall K. Logothetis N. K. (2014). Report dopamine-induced dissociation of BOLD and neural activity in macaque visual cortex. Curr. Biol. 24, 2805–2811. 10.1016/j.cub.2014.10.006 25456449

[B109] Zappe A. C. Pfeuffer J. Merkle H. Logothetis N. K. Goense J. B. M. (2008). The effect of labeling parameters on perfusion-based fMRI in non-human primates. J. Cereb. Blood Flow Metab. 28, 640–652. 10.1038/sj.jcbfm.9600564 17960143

[B110] Zhao F. Jin T. Wang P. Kim S.-G. (2007). Improved spatial localization of post-stimulus BOLD undershoot relative to positive BOLD. Neuroimage 34, 1084–1092. 10.1016/j.neuroimage.2006.10.016 17161623 PMC1876719

[B111] Zong X. Kim T. Kim S.-G. (2012). Contributions of dynamic venous blood volume versus oxygenation level changes to BOLD fMRI. Neuroimage 60, 2238–2246. 10.1016/j.neuroimage.2012.02.052 22401759 PMC3339492

