# Supplementary material for: Determining Excitatory and Inhibitory Neuronal Activity from Multimodal fMRI Data Using a Generative Hemodynamic Model
Source: Front Neurosci. 2017 Nov 10;11:616. doi: 10.3389/fnins.2017.00616 (PMC5715391; doi:10.3389/fnins.2017.00616)
Supplement: Supplementary file 1 [file Table1.docx]

Supplementary Material

Determining excitatory and inhibitory neuronal activity from multimodal fMRI data using a generative hemodynamic model

Martin Havlicek^*^, Dimo Ivanov, Alard Roebroeck, Kamil Uludag

*** Correspondence:** Martin Havlicek: m.havlicek@maastrchtuniversity.nl

# Model parameter assumptions and priors specification

The modeled responses defined in all experiments were fitted to the measured data with observation equations as parameterized in Eq. (1-3) using variational Laplace (VL) optimization algorithm ([Friston et al., 2007](file:///C:\Users\m.havlicek\Dropbox\Copy\Manuscripts_2017\SM.docx#_ENREF_18)). All physiological model parameters, including MR field strength- and MR sequence-specific parameters are specified in Table S1. The parameters that were optimized during model inversion are highlighted with red color and the remaining parameters (in black) were kept constant. As the majority of model parameters are constrained to positive values, they are characterized by log-normal transforms, e.g. $\tau\cdot\exp(\tilde{\tau})$, where $\tilde{\tau}$ represents the latent parameter (being optimized) that scales the default value of the physiological parameter $\tau$. The only parameter that does not have this constraint is the input strength, $c$. This means that it can be either positive or negative. All optimized parameters (i.e. $c$ and latent parameters) are defined in terms of priors described by Gaussian distributions with zero means and non-zero variances (see Table S1). Many parameters were allowed to vary between stimulation period (SP) and post-stimulation period (PSP). To achieve that, latent parameters for SP, e.g. $\tilde{\tau}_{SP}$, were modulated by modulatory input during PSP, $u_{PSP}$, which was scaled by additional latent parameters, e.g. $\tilde{\tau}_{PSP}$. The modulatory input function, $u_{PSP}$, was one during PSP and zero elsewhere. This means that to control certain physiological parameter during the two different time periods, we used the following expression: e.g. $\tau\cdot exp(\tilde{\tau}_{SP}+\tilde{\tau}_{PSP}\cdot u_{PSP})$, see Table S2. Further details on model parameters were described in ([Havlicek et al., 2015](#_ENREF_2)).

**Table S1.** Model parameters and their priors.

|  | Physiological parameters | | | | Latent parameter | | |
| --- | --- | --- | --- | --- | --- | --- | --- |
|  | Parameter | Name | Unit | Value | scaling parameter | mean | Variance |
| *Neuronal model:* | | | | | | | |
|  | $c$ | Input strength | - | 1/16 | - | 0 | 0.1 |
|  | $\sigma$ | Excitatory self-connection | s^-1^ | 0.4 | $\tilde{\sigma}_{x}$ | 0 | $e^{-2}$ |
|  | $\mu$ | Inhibitory-excitatory connection | s^-1^ | 0.2 | $\tilde{\mu}_{x}$ | 0 | $e^{-1}$ |
|  | $\lambda$ | Inhibitory gain factor | s^-1^ | 0.1 | $\tilde{\lambda}_{x}$ | 0 | $e^{-3}$ |
| *NVC:* | | | | | | | |
|  | $\varphi$ | Decay of vasoactive signal | s^-1^ | 0.6 | - | - | - |
|  | $\phi$ | Gain of vasoactive signal | s^-1^ | 1.5 | - | - | - |
|  | $\chi$ | Decay of blood inflow signal | s^-1^ | 0.6 | $\tilde{\chi}_{x}$ | 0 | $e^{-3}$ |
| *Hemodynamic model:* | | | | | | | |
|  | $\alpha$ | Grubb’s exponent | - | 0.3 | - | - | - |
|  | $t_{0}$ | Mean (venous) transit time | s | 2.5 | $h$ | 0 | $e^{-3}$ |
|  | $\tau$ | Viscoelastic time constant | s | 4 | $\tilde{\tau}_{x}$ | 0 | $e^{0}$ |
|  | $E_{0}$ | Resting oxygen extraction fraction | - | 0.35 | - | - | - |
| *BOLD signal:* | | | | | | | |
|  | $V_{0}$ | Resting (venous) blood volume fraction | - | 0.025 | $h$ | 0 | $e^{-3}$ |
|  | $TE$ | Echo-time | ms | *I:* 33  *II:* 15  *III:* 20 | - | - | - |
|  | $\varepsilon$ | Resting intra-to-extravascular signal ratio | - | *I:* 0.7  *II:* 0.05  *III:* 0.35 | - | - | - |
|  | $r_{0}$ | Regression slope of the changes in the intravascular signal relaxation rate with changes in deoxygenated hemoglobin fraction (from 0.4 to 0.3) | s^-1^ | *I:* 50  *II:* 200  *III:* 100 | - | - | - |
|  | $\vartheta_{0}$ | Frequency offset at the surface of a blood vessel for fully deoxygenated blood | s^-1^ | *I:* 80.6  *II:* 252.5  *III:*126.3 | - | - | - |
| *Additional:* | | | | | | | |
|  | $w_{n}$ | Neuronal response scaling parameter |  | 1 | $\tilde{w}_{n,x}$ | 0 | $e^{-2}$ |
|  | $w_{a}$ | Arterial CBV scaling parameter |  | 1 | $\tilde{w}_{a,x}$ | 0 | $e^{-2}$ |
|  | $w_{v}$ | Venous CBV scaling parameter |  | 1 | $\tilde{w}_{n,x}$ | 0 | $e^{-2}$ |
| *I, II, III* – refer to the first, second, and the third experiment acquired at MRI scanner with 3 T, 9.4 T and 4.7 T magnetic field strength, respectively.  Subscript $x$ indicates that the parameter is time-period- or condition-specific. In Table S2, $x$ is replaced by SP or PSP, or/and S (Static) or F (Flickering) or/and P (Positive) or N (Negative). | | | | | | | |

**Table S2.** Model parameters (log-normal) transformations and modulatory inputs assignments.

|  |  | | *Free parameters* | | | | | | | | | | | | | |
| --- | --- | --- | --- | --- | --- | --- | --- | --- | --- | --- | --- | --- | --- | --- | --- | --- |
|  |  | | **Neuronal model** | | | | | | **NVC** | | **Hemodynamic model** | | | **Additional** | | |
|  |  | | $c$ (-) | | $\sigma$ (s^-1^) | | $\mu$ (s^-1^) | $\lambda$ (s^-1^) | | $\chi$ (s^-1^) | $\tau$ (s) | | $t_{0}$ (s), $V_{0}$ (%) | $w_{n}$ (-) | $w_{a}$ (-) | $w_{v}$(-) |
| *Experiment I: CBF & BOLD responses* | | | | | | | | | | | | | | | | |
| SP | | Static | $c_{S}$ | $\sigma\cdot exp(\tilde{\sigma}_{SP,S})$ | | $\mu\cdot exp(\tilde{\mu}_{SP,S})$ | | $\lambda\cdot exp(\tilde{\lambda}_{S})$† | $\chi\cdot exp(\tilde{\chi})$ | | $\tau\cdot exp(\tilde{\tau}_{SP})$ | $V_{0}\cdot exp(h)$,  $t_{0}=V_{0}/F_{0}$ | | - | - | - |
|  |  | Flicker | $c_{F}$ | $\sigma\cdot exp(\tilde{\sigma}_{SP,F})$ | | $\mu\cdot exp(\tilde{\mu}_{SP,F})$ | | $\lambda\cdot exp(\tilde{\lambda}_{F})$‡ |  |  |  |  |  |  |  |  |
| PSP | | Static | - | $\sigma\cdot exp(\tilde{\sigma}_{SP,S}+\tilde{\sigma}_{PSP,S}\cdot u_{PSP})$ | | $\mu\cdot exp(\tilde{\mu}_{SP,S}+\tilde{\mu}_{PSP,S}\cdot u_{PSP})$ | | † |  |  | $\tau\cdot exp(\tilde{\tau}_{SP}+\tilde{\tau}_{PSP}\cdot u_{PSP})$ |  |  |  |  |  |
|  |  | Flicker | - | $\sigma\cdot exp(\tilde{\sigma}_{SP,F}+\tilde{\sigma}_{PSP,F}\cdot u_{PSP})$ | | $\mu\cdot exp(\tilde{\mu}_{SP,F}+\tilde{\mu}_{PSP,F}\cdot u_{PSP})$ | | ‡ |  |  |  |  |  |  |  |  |
| *Experiment II: CBF & total CBV & BOLD responses* | | | | | | | | | | | | | | | | |
| SP | | Gratings | $c$ | $\sigma\cdot exp(\tilde{\sigma}_{SP})$ | | $\mu\cdot exp(\tilde{\mu}_{SP})$ | | $\lambda\cdot exp(\tilde{\lambda})$ | $\chi\cdot exp(\tilde{\chi})$ | | $\tau\cdot exp(\tilde{\tau}_{SP})$ | $V_{0}\cdot exp(h)$,  $t_{0}=V_{0}/F_{0}$ | | - | $w_{a}\cdot exp(\tilde{w}_{a})$ | $w_{v}\cdot exp(\tilde{w}_{v})$ |
| PSP | |  | - | $\sigma\cdot exp(\tilde{\sigma}_{SP}+\tilde{\sigma}_{PSP}\cdot u_{PSP})$ | | $\mu\cdot exp(\tilde{\mu}_{SP}+\tilde{\mu}_{PSP}\cdot u_{PSP})$ | |  |  |  | $\tau\cdot exp(\tilde{\tau}_{SP}+\tilde{\tau}_{PSP}\cdot u_{PSP})$ |  |  |  |  |  |
| *Experiment III: Neuronal & BOLD responses* | | | | | | | | | | | | | | | | |
| SP | | Positive | $c_{P}$ | $\sigma\cdot exp(\tilde{\sigma}_{SP,P})$ | | $\mu\cdot exp(\tilde{\mu}_{SP,P})$ | | $\lambda\cdot exp(\tilde{\lambda}_{P})$† | $\chi\cdot exp(\tilde{\chi}_{P})$† | | $\tau\cdot exp(\tilde{\tau}_{SP})$ | $V_{0}\cdot exp(h)$,  $t_{0}=V_{0}/F_{0}$ | | $w_{n}\cdot exp(\tilde{w}_{n,P})$† | - | - |
|  |  | Negative | $c_{N}$ | $\sigma\cdot exp(\tilde{\sigma}_{SP,N})$ | | $\mu\cdot exp(\tilde{\mu}_{SP,N})$ | | $\lambda\cdot exp(\tilde{\lambda}_{N})$‡ | $\chi\cdot exp(\tilde{\chi}_{N})$‡ | |  |  |  | $w_{n}\cdot exp(\tilde{w}_{n,N})$‡ |  |  |
| PSP | | Positive | - | $\sigma\cdot exp(\tilde{\sigma}_{SP,P}+\tilde{\sigma}_{PSP,P}\cdot u_{PSP})$ | | $\mu\cdot exp(\tilde{\mu}_{SP,P}+\tilde{\mu}_{PSP,P}\cdot u_{PSP})$ | | † | † | | $\tau\cdot exp(\tilde{\tau}_{SP}+\tilde{\tau}_{PSP}\cdot u_{PSP})$ |  |  | † |  |  |
|  |  | Negative | - | $\sigma\cdot exp(\tilde{\sigma}_{SP,N}+\tilde{\sigma}_{PSP,N}\cdot u_{PSP})$ | | $\mu\cdot exp(\tilde{\mu}_{SP,N}+\tilde{\mu}_{PSP,N}\cdot u_{PSP})$ | | ‡ | ‡ | |  |  |  | ‡ |  |  |

**Reference**

Friston, K., Mattout, J., Trujillo-Barreto, N., Ashburner, J., and Penny, W. (2007). Variational free energy and the Laplace approximation. *NeuroImage* 34(1)**,** 220-234. doi: 10.1016/j.neuroimage.2006.08.035.

Havlicek, M., Roebroeck, A., Friston, K., Gardumi, A., Ivanov, D., and Uludag, K. (2015). Physiologically informed dynamic causal modeling of fMRI data. *NeuroImage* 122**,** 355-372. doi: 10.1016/j.neuroimage.2015.07.078.
